# Host-Specific Interplay between Foot-and-Mouth Disease Virus 3D Polymerase and the Type-I Interferon Pathway

**DOI:** 10.3390/v15030666

**Published:** 2023-03-01

**Authors:** Morgan Sarry, Grégory Caignard, Juliette Dupré, Stephan Zientara, Damien Vitour, Labib Bakkali Kassimi, Sandra Blaise-Boisseau

**Affiliations:** 1 UMR VIROLOGIE, INRAE, École Nationale Vétérinaire d’Alfort, ANSES Laboratoire de Santé Animale, Université Paris-Est, 94700 Maisons-Alfort, France; gregory.caignard@vet-alfort.fr (G.C.); juliette.dupre@vet-alfort.fr (J.D.); stephan.zientara@anses.fr (S.Z.); damien.vitour@vet-alfort.fr (D.V.); labib.bakkali-kassimi@anses.fr (L.B.K.); 2 AgroParistech, 16 Rue Claude Bernard, 75005 Paris, France

**Keywords:** protein–protein interactions, virus–host interactions, foot-and-mouth disease virus (FMDV), 3D polymerase, interferon pathway, host specificity

## Abstract

Foot-and-mouth disease (FMD) is a highly contagious viral disease affecting cloven-hoofed animals. One of the issues related to this disease is the persistence of its causative agent, foot-and-mouth disease virus (FMDV). While the mechanisms of FMDV persistence remain unclear, there are clues that it may be related to protein–protein interactions (PPI) between viral proteins and cellular proteins involved in the interferon (IFN) response. Since FMDV persistence has been described in cattle, sheep and goats but not in swine, we screened PPI involving FMDV proteins and sixteen major type-I IFN pathway proteins from these four species by nanoluciferase-2-hybrid complementation assay, in order to identify new PPI and determine their host specificity. As the results concerning the 3D^pol^ were the most interesting in view of the limited data concerning its role in immune escape, we decided to focus particularly on this protein. The identified PPI were confirmed by GST pull-down. We identified PPI between 3D^pol^ and seven IFN pathway proteins, namely, IKKα, IKKε, IRF3, IRF7, NEMO, MDA5 and MAVS. These PPI are conserved among the four studied species, with the exception of the one between 3D^pol^ and MAVS, which was only found with the swine protein. We also showed, using luciferase reporter assays, that 3D^pol^ could inhibit the induction phase of the IFN pathway. These results demonstrate, for the first time, a putative role for 3D^pol^ in FMDV innate immune escape.

## 1. Introduction

Foot-and-mouth disease (FMD) is a highly contagious disease that affects domestic and wild cloven-hoofed animals, in particular livestock such as cattle, sheep, goats, and pigs. It represents a major threat to animal health because its outbreak impact is considerable from both societal and economic points of view. The etiological agent causing FMD is known as foot-and-mouth disease virus (FMDV). This pathogen is a single-stranded positive RNA virus, which belongs to the genus *Aphtovirus* within the *Picornaviridae* family [1]. The FMDV genome exhibits a high mutation rate leading to seven different serotypes, namely, A, O, C, Asia 1, SAT1, SAT2 and SAT3, each subdivided into several subtypes [2]. The FMDV genome contains an open reading frame (ORF) around 7 kb in length, flanked by untranslated regions (5′UTR and 3′UTR). The ORF encodes a unique polyprotein precursor consisting of four structural proteins (VP1, VP2, VP3 and VP4) and eleven non-structural proteins (Lab_pro_, Lb_pro_, 2A, 2B, 2C, 3A, 3B1, 3B2, 3B3, 3C and 3D^pol^) [3]. There are two different forms of the Lpro protein, namely Labpro and Lb_pro_, depending on the start codon from which translation is initiated. Lb_pro_, shorter by 28 amino acids, is the predominant form in vivo. FMDV has three very similar, but not identical, copies of 3B, namely 3B1, 3B2 and 3B3 [4].

FMDV-related clinical symptoms differ between affected species. Generally, they include fever, sudden lameness, lesions on the hooves, tongue and udders and a drop in productivity [5]. After clinical recovery, FMD virus persists beyond 28 days post infection in the pharynx of more than 50% of ruminants without clinical signs, regardless of their specific FMD immune status [6,7]. Over the last few years, this 28-day arbitrary threshold has been controverted. Indeed, according to some experiments, it appears that viral clearance occurs earlier than previously assumed, between 10 to 21 days beyond infection, reflecting the animal’s vaccination status [8,9,10]. Healthy carriers represent a potential threat of transmission of FMDV to susceptible animals and hence an obstacle to successful control of the disease [8,11]. Indeed, it has recently been shown that healthy carriers, who are frequently co-infected, are likely to be the source of inter-typical recombination of FMDV. It has also been demonstrated that these reassortant viruses have a high propensity to escape the immune system, facilitating their spread in the infected population [12,13].

More than 50 years after its first description, the establishment, maintenance and resolution mechanisms of FMDV persistence remain unclear [14]. Among the missing information, there is no explanation as to why FMDV persistence is not described in all susceptible species. For example, FMDV persistence has been reported in cattle and sheep, but not in pigs [15,16]. Many studies have already been carried out to advance knowledge regarding FMDV persistence. Thus, co-evolution between the virus and its host has been demonstrated in hamster kidney cells, as well as in a bovine kidney cell line and pharynx primary cells during a persistent infection [17,18,19]. In addition, modulation of cellular immunity has been associated with FMDV in a bovine in vivo model, while the role of multiple FMDV proteins has been described as being involved in interferon (IFN) response subversion [20,21]. Comparison of transcriptional responses during acute and long-term infection of a bovine primary cell model close to the biological system revealed a diminished IFN response that was ineffective in eliminating the virus during persistent infection [22,23]. Among the hypotheses explored to explain FMDV persistence, modulation of the cellular response, leading to the establishment and maintenance of an equilibrium between the virus and its host via the establishment of protein–protein interactions, seems interesting. Moreover, numerous interactions have already been demonstrated between FMDV and a range of cellular proteins involved in the immune response and, more particularly, in the type-I interferon pathway [21,24,25]. IFN production relies on virus detection by membrane and cytoplasmic sensors. These sensors allow the activation of signalling cascades via the TLR, RLR or NLR pathway, leading to the activation of transcription factors involved in the production of IFN-α/β and pro-inflammatory cytokines in the production phase. Finally, the signalling phase consists of the binding of IFN-a/b to its receptor, which generates an activation signal that propagates in the infected cell and the surrounding ones via the JAK/STAT pathway to express numerous interferon-regulated genes (ISG) and activate the antiviral response. Among the proteins of the type-I IFN pathway, 16 have been shown to be involved in more than 75% of protein interactions between viruses and their hosts (review in preparation). We will therefore focus on these proteins in this study. This study will also explore the study model’s impact, using proteins from four FMD-susceptible species for which persistence of the virus has either been described or not, namely, cattle, sheep, goat and swine.

Like many viruses, FMDV has evolved many strategies for evading the antiviral immune response. Numerous interactions have been identified between FMDV proteins and cellular proteins involved in the innate immune responses of its different hosts. Several reviews have also been written to list these interactions [21,25,26]. According to these reviews, it appears that not all viral proteins are equally important in viral escape from the host’s antiviral action. Indeed, Lpro and 3C seem to be the most important in terms of interaction quantity and diversity, followed by 3A, 2B and 2C. In contrast, proteins 2A, 3B and 3D^pol^ are not acknowledged as importantly involved in immune escape, since no interactions have been described so far for 2A, while very few data are available for FMDV 3B and 3D^pol^. To date, the only known interactions for FMDV 3D^pol^ are its binding with mouse protein Sam68 and swine ATP-dependent RNA helicase DDX1 [27,28].

The protein 3D polymerase (3D^pol^) is the last protein encoded by the FMDV genome. This RNA-dependent RNA polymerase is encoded by a highly conserved region within the FMDV serotypes. It becomes active after 3CD precursor processing and plays a major role in FMDV RNA replication and thus in the viral cycle [29]. As FMDV polymerase is associated with a high error rate due to its low fidelity and lack of proof-reading activity, it contributes to the virus’s high genetic and antigenic diversity [30]. FMDV RNA polymerase catalyses the synthesis of negative-strand genomic intermediates and the subsequent new positive strands in replication organelles derived from the membranes of the endoplasmic reticulum and the Golgi apparatus [31]. To carry out viral replication, 3D^pol^ complexes with other non-structural proteins, such as 2C, 3A, 3B and 3CD precursors or other 3D^pol^ [32,33]. The interaction domains carried by FMDV 3D^pol^, as well as its nuclear localization sequence (NLS), combined with its ability to interact with other viral proteins and the interactions described between other picornavirus 3D^pol^ and cellular proteins, may indicate that the non-catalytic role of FMDV 3D^pol^ could currently be underestimated. Our work has supported this hypothesis by revealing new protein–protein interactions involving FMDV 3D^pol^ and IFN type-I pathway proteins.

## 2. Materials and Methods

### 2.1. Cells

Human embryonic kidney (HEK-293T) cells were maintained in Dulbecco’s modified Eagle’s medium (DMEM) + GlutaMAX™ (Gibco, Grand Island, NY, USA) supplemented with 10% foetal calf serum (FCS, Eurobio Scientific, Les Ulis, France), 100 mg/mL penicillin–streptomycin (Gibco) and 1% sodium pyruvate (Gibco) at 37 °C and 5% CO_2_.

BUcEC, endothelial primary cells derived from bovine umbilical cord, were maintained in improved minimum essential media (OptiMEM) + GlutaMAX™ (Gibco) supplemented with 10% foetal calf serum (FCS, Eurobio), 100 mg/mL penicillin–streptomycin (Gibco) and 100 μg/mL streptomycin and 0.8 μg/mL fungizone (Gibco, ) at 37 °C and 5% CO_2_ [34].

Porcine kidney (PK-15) cells were maintained in Dulbecco’s modified Eagle’s medium (DMEM) + GlutaMAX™ (Gibco) supplemented with 10% foetal calf serum (FCS, Eurobio Scientific), 100 mg/mL penicillin–streptomycin (Gibco), 1% of non-essential amino acids (Gibco) and 1% sodium pyruvate (Gibco) at 37 °C and 5% CO_2_.

### 2.2. Plasmid Libraries

#### 2.2.1. Viral Plasmids Library

The viral plasmids library used in this study was built from FMDV O/FRA/1/2001 Clone 2.2 (GenBank: OV121130.1). Viral RNA was extracted using the QIAamp Viral RNA Mini Kit for purification of viral RNA and the QIAcube^®^ workstation (Qiagen, Hilden, Germany) according to the manufacturer’s recommendations for the manual lysis protocol. Viral RNA was eluted in 60 µL of RNase-free water. Reverse transcription was done using the Transcriptor High Fidelity cDNA Synthesis Kit (Roche, Basel, Switzerland). Briefly, viral RNA was incubated with random hexamer primers (60 µM) for 10 min at 65 °C to denature the RNA and then cooled on ice for 1 min. Transcriptase reaction buffer (1X), RNase inhibitor (20 U), dNTPs (100 μM of each), dTT (50 mM) and Transcriptor High Fidelity reverse transcriptase (22 U) were added to the template–primer mixture. Reverse transcription was performed by a 30 min incubation at 55 °C, then reverse transcriptase inactivation was done by a 5 min incubation at 85 °C. Each viral target of interest was amplified by polymerase chain reaction (PCR) using Q5 Hot Start High Fidelity DNA Polymerase (New England Biolabs, Ipswich, MA, USA) using the primers described in the Supplementary Data (Table S1).

These primers have flanking regions at their 3′ and 5′ terminus, enabling the insertion of each viral sequence into a pDONR207 (Invitrogen, Waltham, MA, USA) using the Gateway^®^ recombination cloning system (Invitrogen). PCR was run with 1 µL of cDNA in Q5 reaction buffer (1X), adding 0.02 U/µL of Q5 Hot Start High Fidelity DNA Polymerase, 200 µM of dNTPs and 0.5 µM of forward and reverse primers. Amplification was performed for 35 cycles as follows: 98 °C for 10 s, 58 °C for 30 s and 72 °C for 2 min, followed by a 10 min final elongation at 72 °C.

All amplified viral sequences were then cloned into pDONR207 by Gateway^®^ cloning technology. A total of 500 ng of pDONR207 vector was incubated overnight at room temperature with 500 ng of PCR product and 2 µL BP Clonase™ to obtain an entry vector. The concentration of each entry vector was measured (NanoDrop™ One, Thermo Fisher Scientific, Waltham, MA, USA), before they were diluted and sent to sequencing for verification of the resulting constructs (Eurofins Genomics). Entry vectors corresponding to Lab_pro_, Lb_pro_, VP4, VP2, VP3, VP1, 2B, 2C, 3A, 3B1, 3B2, 3B3, 3C and 3D^pol^ of FMDV O/FRA/1/2001 Clone 2.2 were obtained. A construct including all three copies of 3B was also prepared and named 3Btotal (3Bt). Only the construct corresponding to protein 2A could not be obtained. This is probably due to the short sequence of the 2A protein, which has a hairpin structure making it difficult to integrate into a plasmid. To overcome this problem, constructs including 2A were made from broader sequences, namely VP1-2A and 2A-2B. The sequences included in these entry vectors were then recombined into various expression vectors related to the methods further used in this study.

Viral sequences cloned into pDONR207 were transferred into a Gateway^®^-compatible destination vector. For this purpose, 500 ng of entry vector was incubated overnight at room temperature with 500 ng of destination vector and 2 µL of LR Clonase™. Thus, viral sequences were included in the nanoluciferase expression plasmids (pDESTN2H-N1, -N2, -C1 and -C2) for the NanoLuc-2-hybrid, GST-tag plasmid (pDEST27) for the GST pull-down and FLAG-tag plasmid (pCI-Neo-3xflag) for the luciferase reporter assays [35].

#### 2.2.2. Cattle, Sheep, Goat and Swine Plasmid Libraries

Entry vectors expressing sixteen proteins of the type-I IFN pathway (IKKα, IKKε, IRF3, IRF7, MDA5, NEMO, MAVS, TRAF3, STING, TYK2, TRIF, STAT1, STAT2, TBK1, PKR and RIG-I) were produced by gene synthesis by service providers (Genecust, Twist Bioscience) from cattle, sheep, goat and swine sequences available in the literature (Genbank references provided in Table S2). Sequences from entry vectors were transferred into a Gateway^®^-compatible destination vector following the same protocol as previously described.

With the exception of the ovine TRIF vector, which could not be successfully re-amplified, all sequences present in these entry vectors were then recombined into various expression vectors relevant to the methods implemented in this study. Thus, these sequences were included in the nanoluciferase expression plasmids (pDESTN2H-N1, -N2, -C1 and -C2) for the NanoLuc-2-hybrid screenings and FLAG-tag plasmid (pCI-Neo-3xflag) for the GST pull-down.

### 2.3. Protein–Protein Interaction (PPI) Assays

#### 2.3.1. Nanoluciferase-2-Hybrid Complementation Assay

Interactions between FMDV O/FRA/1/2001 Clone 2.2. proteins and the sixteen type-I IFN pathway proteins from the four study species were identified by NanoLuc-2-hybrid assay. To maximise the chances of visualising a PPI, we wanted to minimise the steric hindrance impact of the enzyme subunits. Therefore, subunits of nanoluciferase, a small 19.1 kDa protein, were fused to the proteins of interest at their N or C terminus. This screening was performed according to the method established by Choi et al., 2019 to maximise the sensitivity and specificity of the method [35].

HEK293T cells were seeded twenty-four hours before transfection at 100,000 cells per well in 96-well, flat-bottom cell culture microplates (Greiner Bio-One, Frickenhausen, Germany). Plasmid mixes corresponding to the interactions to be tested were made in 96 semi-skirted PCR plates (Thermo Fisher Scientific) by mixing 100 ng of the viral construct carrying one of the two nanoluciferase subunits, 100 ng of the cellular construct carrying the other subunit and 10 ng of Renilla luciferase expression plasmid (pCMV-Luc) used for result normalisation, in a final volume of 15 µL of Jet Prime buffer (Polyplus, Illkirch, France). The Jet Prime solution was prepared by diluting the Jet Prime reagent in Jet Prime buffer at a rate of 0.4 µL Jet Prime and 5 µL buffer per well. This Jet Prime solution was then added to the wells containing the plasmid mixes at a rate of 5.4 µL per well. The 96-well plates were then centrifuged before incubation at RT for 10 min. After incubation, 18 µL of the plasmid–Jet Prime mix was added to the cells.

Forty-eight hours after transfection, the culture medium was removed and 50 μL of passive lysis buffer (Promega, Madison, WI, USA) diluted 1:5 with mili-Q^®^ water (Millipore Corporation, Burlington, MA, USA) was added to each well containing the transfected cells. After 10 min of incubation under maximum agitation at RT, 60 µL of hikarazine-103 (Z103) solution diluted to 1:100 in MES buffer (Promega) were added to each well. Z103 substrate was obtained from Yves L. Janin (Muséum National d’Histoire Naturelle, Paris). This O-acetylated luciferin was hydrolysed prior to its use using a mixture of DMSO and ethanolic hydrochloride as previously described [36,37]. This procedure provided a stock solution of the corresponding luciferin which could be used immediately or stored at −20 °C.

The bioluminescence associated with the enzymatic activity of nanoluciferase was measured using an EnSpire^®^ Alpha luminometer (PerkinElmer, Waltham, MA, USA, 0.1 s integration time). The bioluminescence associated with the enzymatic activity of the Renilla luciferase transfection control was also measured using this instrument after addition of 60 µL of Renilla luciferase Glo substrate (Promega) diluted to 1:100 in Renilla-glow buffer, followed by a 10 min incubation at room temperature sheltered from the light. Positive controls involving the interaction between the human proteins STAT1 and STAT2, which are known to interact very strongly, were performed in each screening to validate the method [38]. Similarly, negative controls involving the absence of interaction between the viral proteins tested and the empty nanoluciferase vectors were performed.

Normalised luminescence ratios (NLR) were calculated using nanoluciferase-complementation-associated luminescence value, negative-control-associated luminescence value and Renilla-luciferase-transfection-control-associated luminescence value. The NLR for a given interacting protein pair A-B was calculated by dividing the luminescent signal by the highest value of luminescence measured in negative controls involving cells co-transfected with viral vectors and nanoluciferase vectors without cellular protein sequences. A second normalisation was performed by dividing the previously obtained results by the Renilla-luciferase-associated luminescence value. To be as stringent as possible, we chose a threshold higher than the usual limit of 3 and set a threshold of NLR equal to 4 in order to discriminate the presence or absence of interaction [39,40]. The choice of this threshold is part of a positive screening approach aimed at reducing the number of proteins targeted for further investigations.

#### 2.3.2. GST Pull-Down Assays

The candidate interactions identified by the NanoLuc-2-hybrid approach were biochemically investigated by GST-pull-down assays. For this purpose, the viral entry vectors were recombined into pDEST27 (Invitrogen) encoding a GST tag, while the vectors containing the cellular protein sequences were recombined into pCI-Neo-3xflag encoding a FLAG tag (kindly provided by Dr Yves Jacob) [41].

Twenty-four hours before transfection, HEK293T cells were seeded at 2,000,000 cells per well in 6-well, flat-bottom cell culture plates (Falcon, Corning, NY, USA). Plasmid mixes corresponding to the interactions to be tested were made by mixing the viral construct carrying a GST tag and the cellular construct carrying a FLAG tag, following the protocol provided by Polyplus. Thirty-six hours post-transfection, the culture medium was removed, and the cells were rinsed and harvested into 1.4 mL of phosphate-buffered saline (PBS, Euromedex, Souffelweyersheim, France). This material was centrifuged for 5 min at 1500× *g*. The supernatants were removed and the pellet resuspended in 300 µL of previously prepared, in-house 120 mM KCl lysis buffer (20 mM MOPS, 120 mM KCl pH 7.4, 2 mM β-mercaptoethanol, 0.5% NP40) and supplemented with anti-protease Complete tablets (Roche) according to the supplier’s recommendations. Cells were lysed on ice for 20 min before being centrifuged for 20 min at 13,000× *g* at +4 °C. The supernatants containing total proteins were then recovered.

A total of 20 µL of these lysates were retained and diluted in 20 µL of 2× loading buffer previously prepared from 4× NuPage Blue (Life Technologies, Carlsbad, CA, USA) diluted 1:2 and 10× reducing agent (Life Technologies) diluted 1:5 in distilled water. The remaining 260 µL of lysates were used for GST pull-down affinity chromatography. For this, the lysates were incubated for 2 h at +4 °C on a slow stirring wheel, in the presence of 500 µL of 120 mM KCl lysis buffer and 35 µL of Sepharose Glutathione beads (GE Healthcare, Chicago, IL, USA) previously washed in 120 mM KCl lysis buffer. The samples were then subjected to a 3-repeat cycle of one-minute centrifugation at 13,000× *g* at +4 °C, removal of the supernatant, washing with 900 µL of 120 mM KCl lysis buffer and 5 min incubation on a slow speed impeller. At the end of these wash cycles, the supernatant was removed, and the pellet re-suspended in 35 µL of 2× loading buffer. The samples (lysates and immunoprecipitation) thus obtained were then used for SDS-PAGE followed by a western blot analysis. For this purpose, 30 µL of these samples and 4 µL of ladder Precision Plus Protein Dual Color Standards (Bio-Rad, Hercules, CA, USA) were deposited on 10-well Bolt Gradient 4–12% Bis-Tris Plus Polyacrylamide Gel (Life Technologies) and separation of the proteins was carried out in Bolt Running Buffer (Life Technologies) diluted 1:20 in distilled water, 10 min at 120 V then 40 min at 180 V. Once migration was complete, the proteins were transferred to a nitrocellulose membrane (Life Technologies) by liquid transfer in Bolt Transfer Buffer (Life Technologies) diluted 1:20 in distilled water with 20% ethanol (Euromedex) and 0.1% antioxidant (Life), for 1 h at 30 V.

The resulting nitrocellulose membranes were saturated by incubation for 30 min in 5% milk solution (Regilait, Saint-Martin-Belle-Roche; France) diluted in PBS-Tween previously prepared by mixing 7.2 L of distilled water, 800 mL PBS 10× and 8 mL of Tween20 (Euromedex). Half of the membranes were then subjected to an anti-FLAG antibody produced in mouse and conjugated with HRP (Sigma-Aldrich, Saint-Louis, MO, USA), used at 1:10,000, while the other half were subjected to an anti-GST antibody produced in rabbit, used at 1:2500. After incubation, the membranes were washed 3 times for 5 min in PBS-Tween buffer. They were incubated for 1 h at RT with an HRP-coupled anti-rabbit secondary antibody (Sigma-Aldrich) used at 1:5000. These membranes were again washed 3 times for 5 min in PBS-Tween buffer. The revelation was performed using a Clarity Western ECL kit (Bio-Rad) at a rate of 3 mL per membrane. The membranes were read using a ChemiDoc device (Bio-Rad). Chemiluminescence acquisition was used to reveal the bands associated with the HRP-coupled antibodies, while colorimetric acquisition was used to reveal the ladder.

Positive controls involving the interaction between FMDV 2C and bovine Beclin1 protein, which are known to interact in a very strong manner, were performed every single time in order to further validate the experiments [42]. Similarly, negative controls involving the cellular proteins to be tested were performed using the GST tag but without any viral protein in order to ensure that the cellular proteins did not aspecifically bind to the Glutathione Sepharose beads. The images obtained in negative were inverted, and brightness and contrast were adjusted using the ImageJ processing freeware.

### 2.4. Luciferase Reporter Assays

The phenotypic impacts of the 3D^pol^ protein of FMDV O/FRA/1/2001 Clone 2.2. and 3D^pol^ protein of FMDV SAT1/KNP/196/91/1 on the type-I IFN pathway were measured by luciferase reporter assays. FMDV 3Bt protein impact was also measured to be used as a control. Two assays were used to study the induction and amplification phases of the pathway, respectively, using HEK293T, BUcEC and PK-15 cells seeded twenty-four hours before transfection at 300,000 cells per well in 24-well, flat-bottom cell culture plates (Falcon).

For the induction phase study, the non-induced control was obtained by mixing 550 ng of pCI-Neo-3xflag, 300 ng of plasmid encoding IFNβ-Firefly luciferase reporter gene (IFN-Beta_pGL3) and 30 ng of pCMV Renilla luciferase. The induction control was obtained by mixing 300 ng of pCI-Neo-3xflag, 300 ng of IFN-Beta_pGL3, 30 ng of pCMV Renilla luciferase and 250 ng of a plasmid (pNΔRIG-I) encoding a constitutively active RIG-I protein. The positive control was achieved by mixing 300 ng IFN-Beta_pGL3, 30 ng of pCMV Renilla luciferase, 250 ng of pNΔRIG-I and 300 ng of a plasmid encoding the fusion protein Non-Structural 3 from bluetongue virus 8 carrying a FLAG tag (BTV8-NS3-3xflag). NS3 of BTV8 is known to be a potent inhibitor of the induction phase of the IFN pathway [43]. The 3D^pol^ protein test was performed by mixing 300 ng IFN-Beta_pGL3, 30 ng of pCMV Renilla luciferase, 250 ng of pNΔRIG-I and 300 ng of a plasmid encoding a fusion protein FMDV 3D^pol^ containing a FLAG tag (destination plasmid pCI-Neo-3xflag). Each condition was performed in triplicate.

For the amplification phase study, non-induced and induction controls were obtained by mixing 300 ng of pCI-Neo-3xflag, 300 ng of plasmid encoding ISRE-Firefly luciferase reporter gene (pISRE-Luc) and 30 ng of pCMV Renilla luciferase. The positive control was achieved by mixing 300 ng of a plasmid encoding the ISRE-luciferase reporter gene, 30 ng of pCMV Renilla luciferase and 300 ng of a plasmid encoding a fusion protein, measles virus V protein carrying a FLAG tag (pMV-V). Measles virus V protein is known to be a potent inhibitor of the signalling phase of the IFN pathway [37]. The 3D^pol^ protein test was performed by mixing 300 ng of plasmid encoding the ISRE-luciferase reporter gene, 30 ng of pCMV Renilla luciferase and 300 ng of a plasmid encoding a fusion protein FMDV 3D^pol^ carrying a FLAG tag. Each condition was performed in triplicate.

The plasmid mixes were systematically incubated for 10 min at RT in the presence of 1 µL of Jet Prime reagent per well. A total of 100 µL of the plasmid–Jet Prime mixes were deposited on the cell monolayers. The induction control, positive control and 3D^pol^ test conditions of the amplification phase assay were treated at twenty-four hours post-transfection with 1000 U of IFN-Beta 1A Human (Tebu-bio, Le Perray-en-Yvelines, France) to trigger the amplification phase of the IFN pathway. Forty-eight hours post-transfection, the culture medium was gently removed and 200 μL of lysis buffer diluted 1:5 with mili-Q^®^ water was added to each well containing the transfected cells. After 10 min of incubation under maximal agitation at RT, 50 µL of each lysate was distributed to two wells of 96-well plates. One of the plates was used for Firefly-luciferase-associated bioluminescence readout after addition of 50 µL per well of Firefly luciferase Bright Glo substrate (Promega). The other plate was used to measure Firefly-luciferase-associated bioluminescence after the addition of 50 µL per well of Renilla luciferase Glo substrate. Both plates were incubated for 5 min at RT in the dark. Bioluminescence readings were taken using an EnSpire^®^ Alpha luminometer. Bioluminescence values associated with Firefly luciferase were normalised by dividing by the bioluminescence value associated with the Renilla luciferase transfection control. The triplicates were then averaged. The percentages of IFN pathway induction were calculated for each condition by dividing the previously normalised bioluminescence values by the mean of the induction controls. The significance of 3D^pol^-associated relative inhibition from the induction control was determined using GraphPad Prism (9.5.0 version, Dotmatics, Boston, MA, USA).

## 3. Results

**Figure 1:**
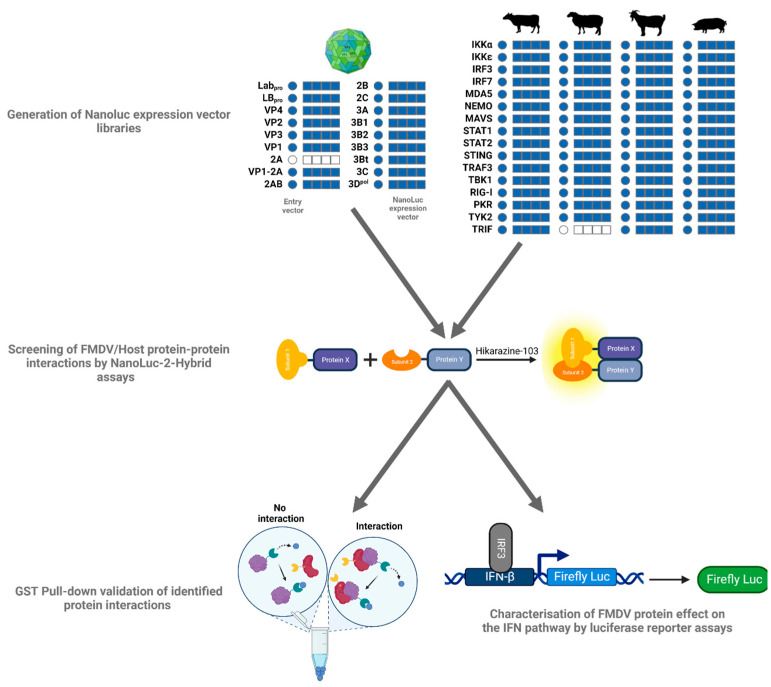
**Identification of PPI between FMDV proteins and major IFN pathway proteins.** NanoLuc expression vector constructs by Gateway^®^ cloning. The circles represent the entry vectors. The squares represent the four configurations of the NanoLuc expression vectors (N1, N2, C1 and C2). In blue: the constructs that were successfully obtained; in white: the constructs that could not be obtained. These vectors were used to perform NanoLuc-2-hybrid screens between 11 FMDV proteins and the 16 IFN pathway proteins selected from the four study species (cattle, sheep, goat and swine). The overall effect of 3Dpol on the IFN pathway was characterised by luciferase reporter assays.

Among the sixty-eight NanoLuc expression vectors obtained by the Gateway^®^ recombination cloning system, forty-four corresponding to Lab_pro_, Lb_pro_, VP3, VP1, 2B, 2C, 3A, 3B1, 3Bt, 3C and 3D^pol^ were used to screen PPI with the sixteen cattle IFN pathway proteins. Altogether, one hundred and seventy-six different interactions were assessed between FMDV proteins and bovine IFN pathway proteins. On the basis of these results, the most interesting viral proteins were used to screen PPI with the sixteen swine IFN pathway proteins. Taking into account the different constructs of the system developed by Choi et al., each interaction was tested under eight combinations, resulting in a total of more than two thousand combinations tested. PPI between the most interesting FMDV proteins and the sixteen selected IFN pathway proteins were screened using the ovine and caprine libraries. The overall effect of viral proteins on the IFN pathway was then characterised by luciferase reporter assays, while the PPI identified by the NanoLuc-2-hybrid assay were challenged by GST pull-down assay (Figure 1).

### 3.1. NanoLuc-2-Hybrid Assays for Binary Protein–Protein Interactions FMDV/Host Screening

#### 3.1.1. Evidence of PPI between FMDV Viral Proteins and Cattle, Sheep, Goat and Swine IFN Proteins

**Figure 2:**
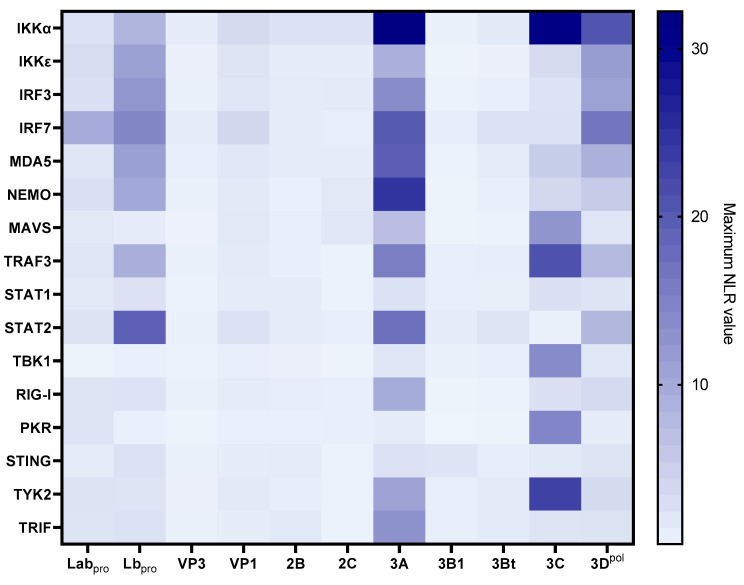
**Screening for protein–protein interactions between eleven FMDV proteins and the sixteen selected IFN pathway proteins from the cattle library**. HEK293T cells were co-transfected with a vector encoding a luminescent protein used for normalisation, as well as one of the four vectors corresponding to each protein, enabling the expression of proteins carrying one of the two nanoluciferase subunits at their N-ter or C-ter extremity. For each pair of interactors, eight combinations were assessed. The bioluminescence values associated with each interactor pair were measured and then normalised in order to limit the biases related to background noise and variations in transfection efficiency. The values provided correspond to the average of three independently performed experiments.

**Figure 3:**
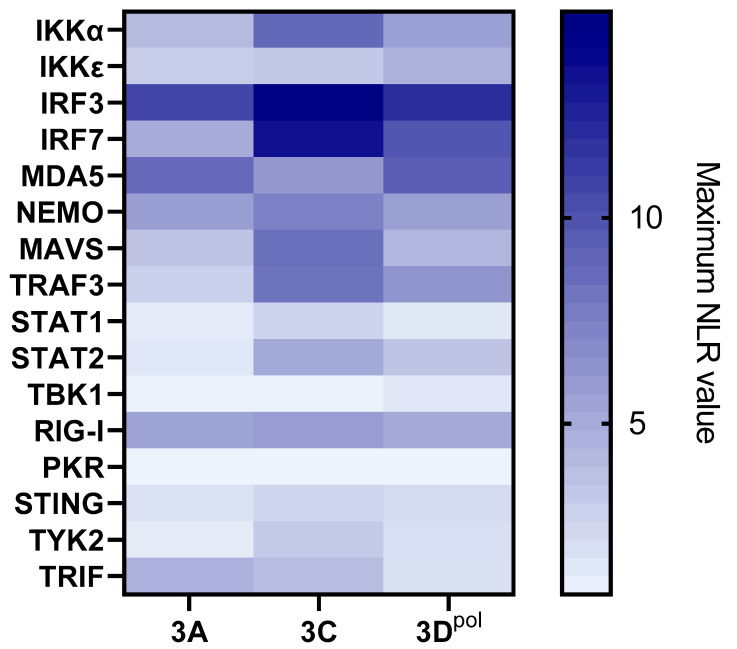
**Screening for protein–protein interactions between FMDV 3A, 3C and 3D^pol^ and the sixteen selected IFN pathway proteins from the swine library**. HEK293T cells were co-transfected with a vector encoding a luminescent protein used for normalisation, as well as one of the four vectors corresponding to each protein, enabling the expression of proteins carrying one of the two nanoluciferase subunits at their N-ter or C-ter extremity. For each pair of interactors, eight combinations were assessed. The bioluminescence values associated with each interactor pair were measured and then normalised in order to limit the biases related to background noise and variations in transfection efficiency. The values provided correspond to the average of three independently performed experiments.

Eleven FMDV O/FRA/1/2001 Clone 2.2 proteins have been tested against the IFN proteins from the cattle library. The overall results of these screenings are presented in Figure 2. The detailed results of each screening are available in the Supplementary Data (Figure S1). The screenings performed with VP3, 2B, 2C, 3B1 and 3Bt did not confirm existing interactions or reveal new ones, as none of the tested interactions was associated with an NLR higher than 4 (Figure S1c,e,f,h,i). The screening involving the VP1 revealed a weak interaction between VP1 and IRF7 (NLR_max_ = 4.22) (Figure 2 and Figure S1d). Screening of PPI between Lab_pro_ and the bovine library highlighted an interaction between Lab_pro_ and IRF7 (NLR_max_ = 10.14) (Figure 2 and Figure S1a). The latter also appeared to interact with the Lpro shorter form (Lb_pro_) in this study. Seven other Lb_pro_ interactors were also identified, namely, STAT2, IRF3, IRF7, MDA5, IKKε, NEMO, TRAF3 and IKKα (Figure 2 and Figure S1b). Interactions involving Lb_pro_ appeared to be relatively important, especially those with STAT2 (NLR_max_ = 19.59) and IRF7 (NLR_max_ = 14.74). Numerous interaction signals have been spotted during 3A protein assays, including IKKα, NEMO, IRF7, MAVS, IRF3, STAT2, TRAF3, MDA5, RIG-I, TRIF, TYK2 and IKKε (Figure 2 and Figure S1g). Very strong interactions have been demonstrated with IKKα (NLR_max_ = 19.59), NEMO (NLR_max_ = 19.59) and IRF7 (NLR_max_ = 19.59). A very strong interaction with IKKα was also revealed with 3C (NLR_max_ = 31.90). Seven additional interactions were revealed using this viral protein, namely TYK2, MDA5, TRAF3, NEMO, TBK1, MAVS and PKR (Figure 2 and Figure S1j). Finally, as shown in Figure 2 and Figure 3, screening performed with the 3D^pol^, for which no interaction with IFN pathway proteins has been described so far, revealed eight potential interactors: IKKα, IRF7, IKKε, IRF3, MDA5, TRAF3, STAT2 and NEMO. The interactions involving IKKα (NLR_max_ = 20.94), IKKε (NLR_max_ = 12.03), IRF3 (NLR_max_ = 11.24) and IRF7 (NLR_max_ = 16.83) were again particularly strong.

As the assays based on the viral proteins Lb_pro_, 3A, 3C and 3D^pol^ were the most interesting in terms of the quantity of potential interactions revealed and in view of the significant variability observed within the Lb_pro_ assays, it was decided to pursue the investigations only on the 3A, 3C and 3D^pol^ proteins.

The three selected proteins were used to screen the swine protein library. The overall results of these screenings are presented in Figure 3. The detailed results of each screening are available in the Supplementary Data (Figure S2). The assays performed with 3A revealed interactions with IRF3, MDA5, NEMO, RIG-I, TRIF, IKKα and IRF7 (Figure 3 and Figure S2a). 3A-IRF3 interaction (NLR_max_ = 10.81) is among the most important interactions observed in this screening, followed by 3A-MDA5 (NLR_max_ = 6.38) and 3A-NEMO (NLR_max_ = 5.87). Screening of 3C evidenced interactions between this protease and swine IRF3, IRF7, MAVS, TRAF3, IKKα, IKKε, STAT2, MDA5, RIG-I and TYK2 as well as with NEMO (Figure 3 and Figure S2b). According to the screening results, the interactions between 3C and IRF3 (NLR_max_ = 15.00) as well as IRF7 (NLR_max_ = 13.63) are the stronger ones. Screening of 3D^pol^ against the porcine library highlighted numerous interactors, such as IRF3, IRF7, MDA5, NEMO, MAVS, IKKα, TRAF3, RIG-I and IKKε (Figure 3). Similarly to 3C, the strongest interactions from these screens involve the swine proteins IRF3 (NLR_max_ = 12.09) and IRF7 (NLR_max_ = 9.89). MDA5 (NLR_max_ = 9.53) also appears to be an important 3D^pol^ interactor.

As 3D^pol^ is particularly interesting in regard to its little-known role in the molecular interplay with the IFN pathway, and as the associated screenings are the least prone to high variability, it was decided in the context of this study to continue the experiments exclusively on this protein.

**Figure 4:**
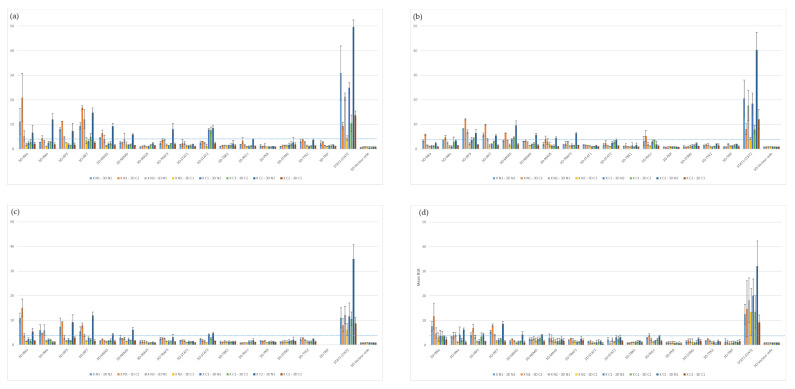
**Screening for protein–protein interactions between FMDV 3D^pol^ and the sixteen selected IFN pathway proteins from the cattle, sheep, goat and swine libraries**. HEK293T cells were co-transfected with a vector encoding a luminescent protein used for normalisation, as well as one of the four vectors corresponding to each protein, enabling the expression of proteins carrying one of the two nanoluciferase subunits at their N-ter or C-ter extremity. For each pair of interactors, eight combinations were assessed. The bioluminescence values associated with each interactor pair were measured and then normalised in order to limit the biases related to background noise and variations in transfection efficiency. The bioluminescence values associated with the human STAT1–STAT2 interaction serve as a positive control, while those associated with the empty 3D^pol^–vector pairs constitute a negative control, indicative of background. A threshold of NLR = 4 was defined to discriminate the interactions that were considered positive. The values provided correspond to the average of three independently performed experiments. (**a**) Screening for protein–protein interactions between FMDV 3D^pol^ and the sixteen selected IFN pathway proteins from the cattle library. (**b**) Screening for protein–protein interactions between FMDV 3D^pol^ and the sixteen selected IFN pathway proteins from the swine library. (**c**) Screening for protein–protein interactions between FMDV 3D^pol^ and the sixteen selected IFN pathway proteins from the sheep library. (**d**) Screening for protein–protein interactions between FMDV 3D^pol^ and the sixteen selected IFN pathway proteins from the goat library.

The 3D^pol^ protein was used to screen the sheep protein library (Figure 4c). This revealed interactions between 3D^pol^ and sheep IKKα, IRF7, IRF3, IKKε, NEMO, STAT2 and MDA5. Interactions involving IKKα (NLR_max_ = 15.07), IRF3 (NLR_max_ = 9.34) and IRF7 (NLR_max_ = 11.95) appear to be particularly important in these screens. As a reminder, the absence of a TRIF-associated vector construct in the ovine library (due to technical issues) does not allow us to conclude on a potential interaction with FMDV 3D^pol^.

Similarly, the 3D^pol^ protein was used to screen the goat protein library (Figure 4d). This time, eight interactors were identified, namely IKKα, IRF7, IRF3, IKKε, MDA5, RIG-I and NEMO. Similar to the screenings against the sheep protein library, the goat proteins IKKα (NLR_max_ = 11.75), IRF3 (NLR_max_ = 8.65) and IRF7 (NLR_max_ = 6.95) appear to interact most strongly with 3D^pol^.

#### 3.1.2. Screening Comparison between the Different Protein Libraries Derived from Four FMD-Susceptible Species

**Figure 5:**
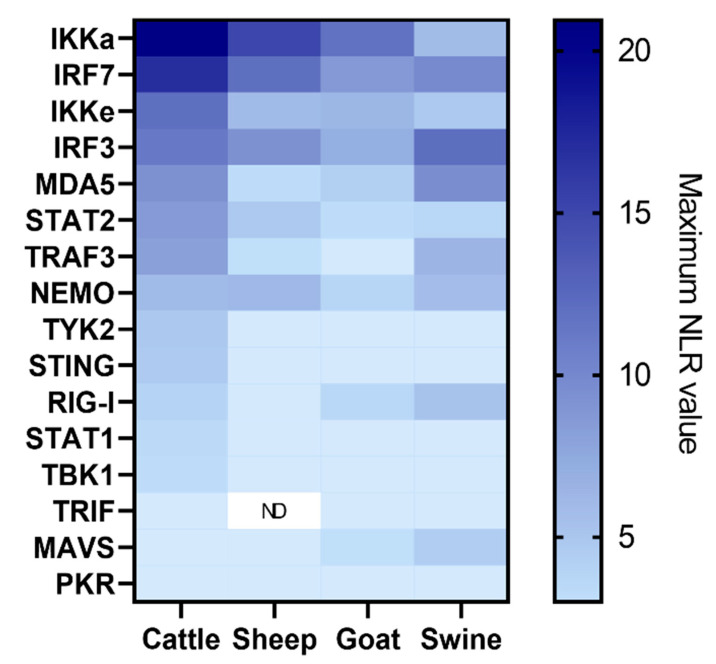
**Heatmap comparing the relative strengths associated with each interaction tested by NanoLuc-2-hybrid screening between FMDV 3D^pol^ and the sixteen selected IFN pathway proteins from the cattle, sheep, goat and swine libraries**. HEK293T cells were co-transfected with a vector encoding a luminescent protein used for normalisation, as well as one of the four vectors corresponding to each protein, enabling the expression of proteins carrying one of the two nanoluciferase subunits at their N-ter or C-ter extremity. For each pair of interactors, eight combinations were assessed. The bioluminescence values associated with each interactor pair were measured and then normalised in order to limit the biases related to background noise and variations in transfection efficiency. Here, only the maximum NLR values have been considered to rank the interactions’ relative strength. The values provided correspond to the average of three independently performed experiments.

Results from the 3D^pol^ screening with the cattle, swine, sheep and goat protein libraries were compared to construct the following heatmap (Figure 5). It seems obvious that a majority of the interactions are conserved among the tested species. Thus, bovine, porcine, ovine and caprine versions of IKKα, IKKΕ, IRF3, IRF7, MDA5 and NEMO interact with 3D^pol^. STAT2-3D^pol^ interaction is evidenced through cattle and sheep IFN pathway proteins screenings, but not in goat and swine. The interaction between 3D^pol^ and TRAF3 is identified using the bovine and swine libraries, while the interaction between 3D^pol^ and RIG-I is identified using the goat and swine libraries. We also evidenced that only the porcine version of MAVS interacts with FMDV polymerase (NLR_max_ = 4.45). Although most of the interactions appear to be conserved within the study species, it is interesting to consider the relative importance that these interactions may have in each model used. Thus, a heatmap showing these interactions’ relative strengths in the different 3D^pol^ screenings is presented to best visualise the findings (Figure 5). The maximum NLR values associated with each tested interactor were used to build this figure.

It appears that, while the relative importance of some interactors, such as IRF7, the second-highest NLR value in the four screens, appears to be conserved across species, this is not the case for all. In particular, significant disparities appear to exist between the cattle, sheep and goat versions of the screening compared to those involving swine proteins. For example, IKKα is the interactor associated with the highest NLR value for the bovine (NLR_max_ = 20.94), ovine (NLR_max_ = 15.07) and caprine (NLR_max_ = 11.75) models, but only ranks fifth for the porcine model (NLR_max_ = 5.82). The same applies to IKKε, which is among the five most important interactors in ruminants but only has the eighth-highest NLR value in swine, or STAT2, which is not among the eight-highest NLR values in the swine library screens, in contrast to the other ones tested. Conversely, IRF3, which appears to be the most important interactor in pigs, is only the third-most important among ruminants. The interaction between RIG-I and 3D^pol^ also appears to be relatively more important in swine than in other species.

### 3.2. FMDV 3D^pol^ Inhibits the IFN Pathway Induction Phase

The significant number of 3D^pol^ protein interactors identified in this study among the major type-I IFN pathway proteins suggests that this viral polymerase has an impact on the pathway. To further investigate the effect of PPI between FMDV 3D^pol^ and type-I IFN pathway proteins, relevant luciferase reposrter assays were therefore performed, the first focusing on the induction phase, upstream of IFN production, and the second focusing on the amplification phase, downstream of IFN production. In order to determine the associated interserotypic variability, we have decided to do such an experiment with type O FMDV 3D^pol^, as well as type SAT1 FMDV 3D^pol^.

#### 3.2.1. 3D^pol^ Effects on the IFN Pathway Induction Phase

**Figure 6:**
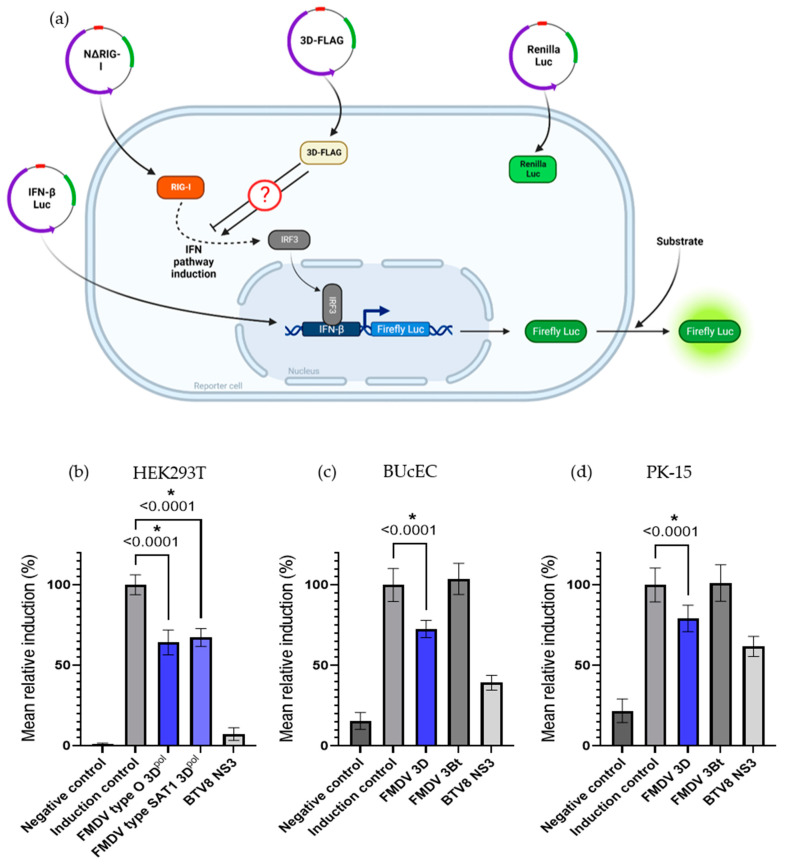

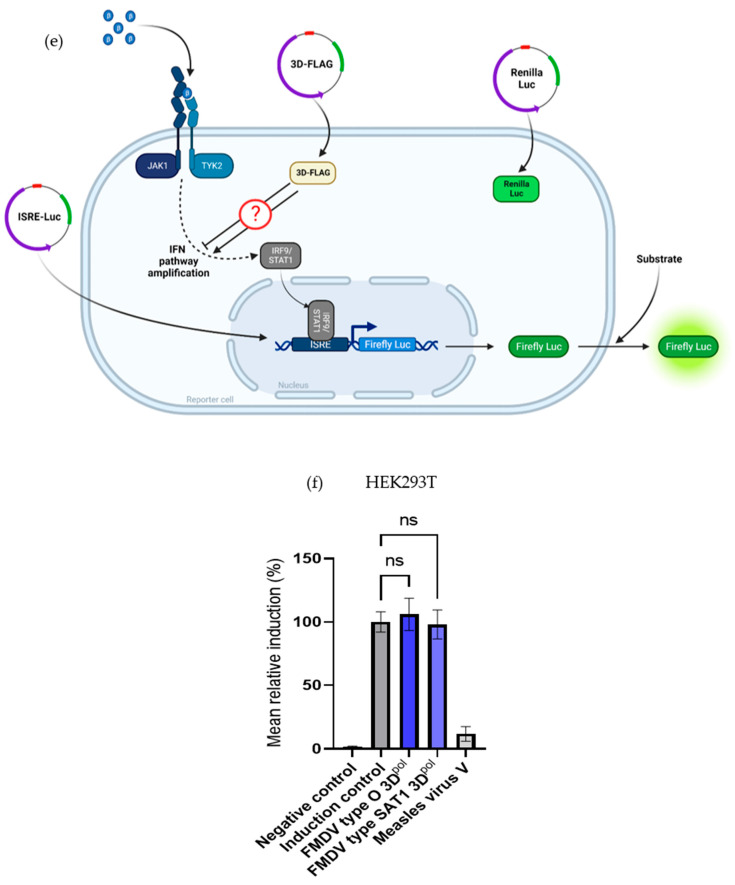
**Investigating the effect of FMDV 3Dpol on the IFN pathway.** (**a**) **Principles of luciferase reporter assays, focusing on the effect of FMDV 3Dpol on the IFN pathway induction phase.** HEK293T, BUcEC and PK-15 cells were co-transfected with a vector encoding an IFN-β-luciferase reporter gene, a vector responsible for the expression of a constitutively activated RIG-I protein, a vector encoding a FLAG-tagged FMDV 3Dpol and a normalisation vector. The activated RIG-I protein triggers IFN pathway induction, leading to the activation of transcription factors such as IRF3. These transcription factors induce the expression of the reporter gene, resulting in luminescent protein expression. The addition of a substrate allows this luminescence to be read out. After comparison with controls, the luminescence quantified in presence of FMDV 3Dpol indicates whether FMDV 3Dpol has an effect on the IFN pathway induction phase and whether its effect is more inhibitory or activating of this pathway. Created with BioRender.com (accessed on 11 October 2022). (**b**) **Effect of FMDV 3Dpol on the IFN pathway induction phase in HEK293T cells.** Forty-eight hours after transfection, cells were lysed, and the bioluminescence was quantified. Reporter-gene-associated luminescence values were normalised regarding normalisation-protein-associated luminescence values, as well as the induction-control-associated values. Results are expressed as a percentage of relative induction compared to the induction control. Here are represented the average results from six independent experiments, each including three technical replicates. An ANOVA test was performed (FMDV O 3Dpol and FMDV SAT1 3Dpol *p*_value_ < 0.0001). (**c**) **Effect of FMDV 3Dpol on the IFN pathway induction phase in BUcEC cells.** Forty-eight hours after transfection, cells were lysed, and the bioluminescence was quantified. Reporter-gene-associated luminescence values were normalised regarding normalisation-protein-associated luminescence values, as well as the induction-control-associated values. Results are expressed as a percentage of relative induction compared to the induction control. Here are represented the average results from six independent experiments, each including three technical replicates. An ANOVA test was performed (FMDV O 3Dpol *p* value < 0.0001 and FMDV O 3Bt non-significant *p* value = 0.30). (**d**) **Effect of FMDV 3Dpol on the IFN pathway induction phase in PK-15 cells.** Forty-eight hours after transfection, cells were lysed, and the bioluminescence was quantified. Reporter-gene-associated luminescence values were normalised regarding normalisation-protein-associated luminescence values, as well as the induction-control-associated values. Results are expressed as a percentage of relative induction compared to the induction control. Here are represented the average results from six independent experiments, each including three technical replicates. An ANOVA test was performed (FMDV O 3Dpol *p* value < 0.0001 and FMDV O 3Bt non significative *p* value = 0.99). (**e**) **Principles of luciferase reporter assays, focusing on the effect of FMDV 3Dpol on the IFN pathway amplification phase.** HEK293T cells were co-transfected with a vector responsible for the expression of an ISRE-luciferase reporter gene, a vector encoding a FLAG-tagged FMDV 3Dpol and a normalisation vector. Cells were then treated with recombinant IFNβ in order to trigger the JAK/STAT pathway, leading to the activation of transcription factors such as IRF9 and STAT1/2. These transcription factors induce the expression of the reporter gene, resulting in luminescent protein expression. The addition of a substrate allows this luminescence to be read out. After comparison with controls, the luminescence quantified in presence of FMDV 3Dpol indicates whether FMDV 3Dpol has an effect on the IFN pathway amplification phase and whether its effect is more inhibitory or activating of this pathway. Created with BioRender.com (accessed on 11 October 2022). (**f**) **Effect of FMDV 3Dpol on the IFN pathway amplification phase.** Forty-eight hours after transfection, cells were lysed, and the bioluminescence was quantified. Reporter-gene-associated luminescence values were normalised regarding normalisation-protein-associated luminescence values, as well as the induction-control-associated values. Results are expressed as a percentage of relative induction compared to the induction control. Here are represented the average results from six independent experiments, each including three technical replicates. An ANOVA test was performed (FMDV O 3Dpol *p*_value_ = 0.18 and FMDV SAT1 3Dpol *p*_value_ = 0.94). * indicates a statistically significant *p*-value, ns indicates a non-significant *p*-value.

To assess the ability of 3D^pol^ to interfere in the IFN induction phase, luciferase reporter assays have been performed in HEK 293T cells as described in Figure 6a. The controls used validated these luciferase reporter assays, as the induction control resulted in bioluminescence values approximately a hundred times stronger than the background associated with the method (Figure 6b). In addition, inhibition control using the BTV8 NS3 protein also worked, with a reduction of over 90% in the induction of the IFN pathway compared to the induction control. Finally, this test showed an almost 40% reduction in the signal when FMDV 3D^pol^ is expressed. This indicates that, under these experimental conditions, FMDV O/FRA/1/2001 Clone 2.2. 3D^pol^ (36% inhibition) as well as FMDV SAT1/KNP/196/91/1 3D^pol^ (34% inhibition) have an inhibitory activity on the IFN pathway activation phase. After statistical analysis of the data set, it appears that this inhibitory effect is significant.

In view of these results in a cell model particularly suited to the implementation of luciferase reporter assays but not really more relevant to studying FMDV, similar assays were performed in cells described as susceptible to the virus, namely BUcEC and PK-15. In these reporter assays, the induction control resulted in bioluminescence values approximately five times stronger than the background associated with the method (Figure 6c,d). Inhibition control using the BTV8 NS3 protein was associated with a respective reduction of almost 60% and 40% in the induction of the IFN pathway compared to the induction control. When the FMDV type O 3D^pol^ was expressed, the luminescence signal was reduced by nearly 27% in BUcEC and 21% in PK-15. Furthermore, when the FMDV type O 3Bt was expressed, the luminescence signal did not change significantly compared to the induction control.

#### 3.2.2. 3D^pol^ Effects on the IFN Pathway Amplification Phase

In order to study the impact of 3D^pol^ on the IFN pathway amplification phase, another luciferase reporter assay was performed, as described in in Figure 6e. The controls carried out for this test validated the method used. Indeed, the bioluminescence values associated with the induction control of the amplification phase were more than sixty times higher than those associated with the background (Figure 6f). IFN amplification phase inhibition control using measles virus protein V resulted in an almost 90% reduction in IFN pathway induction compared to the induction control. In contrast to luciferase reporter assays that focus on the induction phase of the IFN pathway, this assay did not demonstrate a significant effect of 3D^pol^ on the amplification phase of the IFN pathway under the experimental conditions.

Based on these assays, the 3D^pol^ of FMDV O/FRA/1/2001 Clone 2.2 and SAT1/KNP/196/91/1 appear to be able to inhibit the induction phase of the IFN pathway but do not appear to have an effect on the amplification phase. Furthermore, under these experimental conditions, no significant difference could be observed between 3D^pol^ from FMDV O/FRA/1/2001 Clone 2.2 and FMDV SAT1/KNP/196/91/1.

### 3.3. Biochemical Assessment of Candidate Protein–Protein Interaction by GST-Pull Down

**Figure 7:**
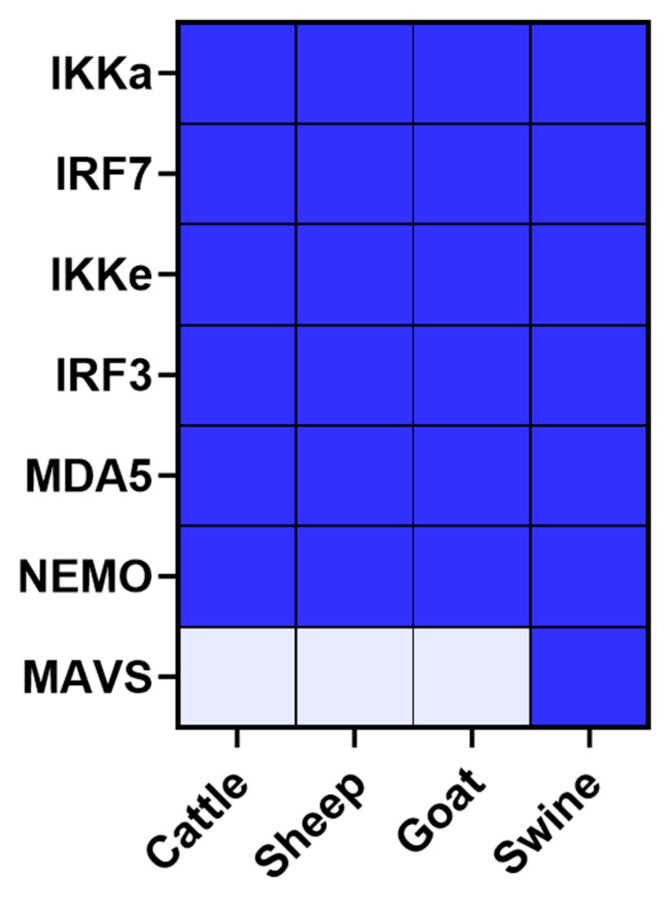
**Overview of GST pull-down validation of previously identified protein–protein interactions between FMDV 3Dpol and seven IFN pathway proteins derived from cattle, sheep, goat and swine.** Protein–protein interaction involving FMDV 3Dpol were assessed by GST pull-down. HEK293T cells were co-transfected with one vector encoding FMDV protein fused to a GST tag, or a control GST, and one vector encoding IFN pathway protein fused to a FLAG tag. In blue, the interactions that were identified by GST pull-down. In white, the interactions not found by GST pull-down.

In order to confirm the interactions, highlighted by the NanoLuc-2-hybrid approach and strengthened by the luciferase reporter assays, these were biochemically confirmed by GST pull-down affinity chromatography. In this assay, interaction between FMDV 3D^pol^, fused to a GST tag, and proteins involved in the IFN pathways from the four study species, fused to a FLAG tag, results in their co-precipitation. Interactor couples to be tested were incubated on Glutathione Sepharose beads. GST tag affinity for these beads allowed GST-fusion proteins to be bound to the beads. When physical interaction exists between the GST-fusion protein and another protein, the latter is also indirectly bound to the beads. After successive washes, the remaining proteins were then unbound to the beads in order to perform an SDS-PAGE followed by a western blot analysis. These analyses were performed on both post-affinity chromatography samples, called IP, and pre-affinity chromatography samples, called Input. Twenty-eight interactions were thus tested, involving FMDV polymerase and cattle, sheep, goat and pig versions of the proteins IKKα, IKKε, IRF3, IRF7, MDA5, NEMO for which all four forms tested were shown to interact with 3D^pol^ by NanoLuc-2-hybrid assay, and MAVS for which only the porcine form appeared to interact with 3D^pol^. All results have been summarised in the figure below (Figure 7).

**Figure 8:**
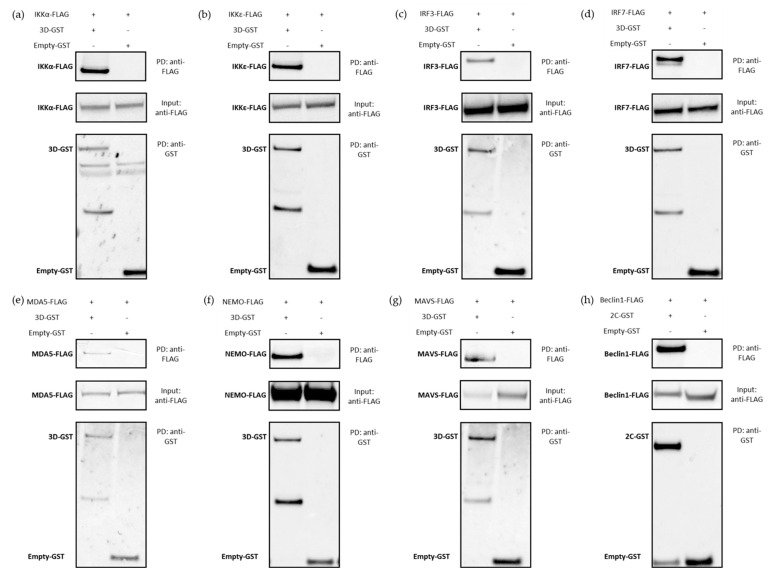
**GST Pull-down validation of previously identified protein–protein interactions between FMDV 3Dpol and seven IFN pathway proteins derived from swine.** Protein–protein interactions involving FMDV 3Dpol were assessed by GST pull-down. HEK293T cells were co-transfected, with one vector encoding FMDV protein fused to a GST tag or a control GST, and one vector encoding IFN pathway protein fused to a FLAG tag. Thirty-six hours post-transfection, the cells were lysed, and the total proteins fraction, corresponding to the “input”, was collected. A part of this fraction was used to perform pull-down assays on Glutathione Sepharose beads. Proteins collected after pull-down were defined as the “PD” condition. Both “input” and “PD” proteins were used to perform western blot analysis. FMDV proteins were identified using an anti-GST antibody. IFN pathway proteins were identified using an anti-FLAG antibody. (**a**) Assessment of 3Dpol and IKKα interaction. (**b**) Assessment of 3Dpol and IKKε interaction. (**c**) Assessment of 3Dpol and IRF3 interaction. (**d**) Assessment of 3Dpol and IRF7 interaction. (**e**) Assessment of 3Dpol and MDA5 interaction. (**f**) Assessment of 3Dpol and NEMO interaction. (**g**) Assessment of 3Dpol and MAVS interaction. (**h**) Positive control involving the interaction between FMDV 2C and the bovine Beclin1 protein, which are known to interact in a very strong manner.

**Figure 9:**
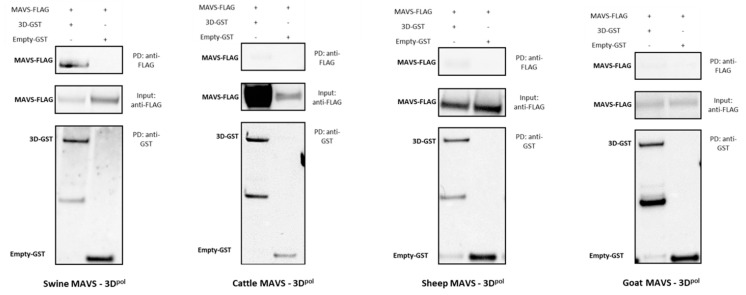
**GST pull-down validation of previously identified host-specific interactions between FMDV 3Dpol and cattle, sheep, goat and swine MAVS.** Protein–protein interactions involving FMDV 3Dpol were assessed by GST pull-down. HEK293T cells were co-transfected with one vector encoding 3Dpol fused to a GST tag and one vector encoding MAVS fused to a FLAG tag. Thirty-six hours post-transfection, the cells were lysed, and the total proteins fraction, corresponding to the “input”, was collected. A part of this fraction was used to perform pull-down assays on Glutathione Sepharose beads. Proteins collected after pull-down were defined as the “PD” condition. Both “input” and “PD” proteins were used to perform western blot analysis. 3Dpol was identified using an anti-GST antibody. MAVS was identified using an anti-FLAG antibody.

Only the western blot analysis resulting from the assays using the swine forms of the IFN pathway proteins are presented in this section (Figure 8). The other western blot analysis are available as Supplementary Data (Figure S3). Despite some disparities regarding visualised band intensity, all overexpressed proteins were detected in the inputs. Anti-FLAG immunoblotting revealed bands corresponding to cellular proteins, and anti-GST immunoblotting revealed viral protein or empty-GST associated bands. (Figure 8 and Figure S3a–c)-Input anti-FLAG and anti-GST). For the pull-down (PD) samples, 2C-GST, 3D-GST and empty-GST bands were detected. Except for the empty-GST control involving the bovine version of NEMO, the affinity chromatography step is validated for all tested conditions (Figure 8 and Figure S3a–c)-PD anti-GST and Input anti-GST). The empty GST-protein interaction controls did not show aspecific binding of IKKα, IKKε, IRF3, IRF7, MDA5 and MAVS proteins (Figure 8 and Figure S3a–c)-PD anti-FLAG). In contrast, weak bands associated to the goat and sheep versions of NEMO were observed among the negative controls. It suggests that these proteins partly binds in a non-specific manner to the Glutathione Sepharose beads or to the GST tag alone, even in absence of the 3D^pol^ protein. As this aspecific binding is less important than the specific binding, it is nevertheless possible to conclude to an interaction between 3D^pol^ and NEMO. With regard to the interaction tests between these IFN pathway proteins and the 3D^pol^ FMDV, bands related to the different proteins studied could be visualised after labelling the PD samples with a FLAG antibody (Figure 8 and Figure S3a–c)-PD anti-FLAG). Bands matching IKKα and IKKε proteins were observed. The same was true for IRF7, whose post-pull-down presence was revealed. Bands corresponding to IRF3, NEMO and MDA5 were also found. These signals, alongside the lack of a specific physical interaction between the GST vector itself and the proteins IKKα, IKKε, IRF3, IRF7, MDA5 and NEMO, suggest that there are effective interactions between these cellular proteins and FMDV 3D^pol^. In contrast, while the interaction assay between 3D^pol^ and the swine MAVS protein showed a band corresponding to MAVS, no signal consistent with this protein was observed for the interaction tests conducted with its bovine, ovine and caprine versions (Figure 9).

Overall, the GST pull-down affinity chromatography tests carried out in this study thus confirmed a large part of the potential interactions highlighted by the NanoLuc-2-hybrid approach and thus confirmed the choice made concerning the positivity threshold set for these screens. Under the experimental conditions tested, 3D^pol^ interacts with bovine, ovine, caprine and porcine forms of IKKα, IKKε, IRF3, IRF7, NEMO and MDA5. Lastly, it was confirmed that only the swine form of MAVS protein interacts with the FMDV polymerase.

## 4. Discussion

Virus survival depends on its ability to adapt to its host in order to facilitate replication and evade the immune response. To do so, viruses manipulate the host’s cellular machinery and highjack natural cellular pathways. These cellular manipulation mechanisms can notably rely on protein interactions between the virus and its host. Thus, through this study, we report that FMDV 3D polymerase is able to interact with several proteins involved in the innate immune response, specifically in the type-I IFN pathway.

Among the numerous interactions highlighted by the NanoLuc-2-hybrid assays are some confirmed interactions already described in the literature (Table 1). Indeed, the interactions between Lb_pro_ and TRAF3 and MDA5, and between 3A and MAVS, MDA5 and RIG-I, already shown in a human model, were confirmed from their bovine versions for the first ones, and from bovine and porcine versions for the three others [44,45,46,47]. The same is true for the 3C–MDA5, 3C–NEMO, Lb_pro_–STAT1/2 and Lab_pro_–IRF7 interactions, which were already demonstrated using porcine proteins and which were found in both the screenings carried out within this study using the porcine and bovine libraries [48,49,50,51]. While the interaction described between Lab_pro_ and swine IRF3 was not found using its bovine version, bovine IRF3 was identified as interacting with the alternative leader protein form Lb_pro_ [50]. It would therefore appear that these interactions are conserved between the different study species.

However, these tests did not confirm all of the interactions already described between FMDV and the IFN pathway players. This could be explained partly by the non-conservation of these interactions, which was highlighted by the use of proteins from different species. For example, the interaction already documented between 3B and the swine version of RIG-I could not be confirmed in the assay using the bovine version [54]. Similarly, it was not possible to confirm the interaction between RIG-I and 2B in the screening based on the cattle library [53]. It is therefore quite conceivable that these interactions could also have been revealed by testing the pig version of this sensor. The situation is exactly the same for the interactions described between Lab_pro_ and the swine forms of TBK1 and MAVS, interactions that were not found with their bovine version [57]. The interactions Lb_pro_–TBK1, Lb_pro_–RIG-I, 2B–MDA5, VP3–MAVS and VP1–MAVS could not be confirmed either [46,52,58,59]. These interactions, which were identified from the human versions of the proteins mentioned, with the exception of VP1–MAVS for which the origin of MAVS is not mentioned, do not seem to be conserved in cattle. As expected, phylogenetic analysis of proteins from the different study species showed significant differences between FMDV-susceptible species and humans. It is thus quite possible that the interactions observed between the virus and human proteins are not conserved in ruminants, and in this case, cattle.

The previously described interactions between 3C and the STAT1 and STAT2 proteins are somewhat different from the other cases mentioned [55]. Thus, even if these interactions highlighted from a human model could not be confirmed through the bovine library screening, it is not possible to conclude that they are not conserved, since it was shown that these interactions were indirect and dependent on co-factors. Without a physical interaction between the proteins tested, the nanoluciferase subunits cannot reassemble and no signal can be detected. The interaction between 3C and STAT2 observed in the porcine library screening, reflecting a physical interaction between the two parts, cannot therefore correspond to the PPI already described. Similarly, the interaction described between 3C and the swine PKR was not found in the porcine assay, which is consistent with the indirect nature of this interaction leading to the 3C-mediated lysosomal degradation of PKR [56]. The interaction found with the bovine version of PKR cannot therefore be related to the interaction described above.

The conclusions concerning the conservation or not of the interactions already described in the literature thus seem to depend on various factors. In view of the results obtained during our screening, it seems relevant to consider the importance of the models used for the identification of PPI, since some of them do not seem to be conserved from one species to another. This problem seems all the more important as a large proportion of the PPI identified were obtained with expressed proteins that were not derived from species sensitive to FMD, such as humans, a species that is not necessarily the most suitable for studying a virus affecting artiodactyls [26]. Furthermore, it seems that other variables also have a role to play. Indeed, the choice of the method appears to be decisive, since, in the case of the NanoLuc-2-hybrid approach, it is only possible to detect interactions if they involve physical proximity of the two partners [35]. Indirect PPI are therefore not detectable by this type of method. Conversely, it is likely that the methods used in this study, involving overexpressed proteins, induce PPI that may not exist between endogenous proteins in an infectious context. As with the species used to obtain the cellular proteins, the FMDV strain used differs from one study to another, and this choice could have a major impact on the PPI detected, particularly those involving the least conserved viral sequences.

Indeed, due to mutations, some strains will affect certain species to a greater or lesser extent. In particular, it was shown that the strain O/SKR/01/2014, containing a mutation-deleting copy of 3B1, was shown to be relatively non-virulent in pigs [60]. Furthermore, it was shown that a single mutation in the 3A sequence permitted an FMDV strain to infect guinea pigs, a non-natural host for the virus, whereas other deletions and substitutions in the 3A sequence can result in virus attenuation and reduced replication efficiency in cattle [61]. The impact of these mutations on the existing PPI between the virus and its hosts could help explain this phenomenon. It would therefore be extremely interesting to combine interaction tests including cell proteins from different susceptible species and viral proteins from different FMDV strains.

Our screenings also revealed new potential interactions involving the proteins VP1, Lab_pro_, Lb_pro_, 3A, 3C and 3D^pol^. According to cattle screening, it was shown that VP1 interacts with IKKα and IRF7, while Lb_pro_ interacts with STAT2, IRF7, IKKε, NEMO, TRAF3 and IKKα. New bovine interactors were also identified for 3A and 3C, respectively, IKKα, NEMO, IRF7, STAT2, TRAF3, TRIF, TYK2 and IKKε and IKKα, TYK2, TRAF3, TBK1, MAVS and IKKε. Meanwhile, numerous interactions were found for 3D^pol^ with IKKα, IRF7, IKKε, IRF3, MDA5, TRAF3, STAT2, NEMO, RIG-I and TYK2. Concerning proteins 3A and 3C, while five of their newly identified interactions with the bovine library are respectively conserved among the porcine library, it appears that some swine proteins do not interact with these two viral proteins. For example, no interactions could be identified between 3A and STAT2, TRAF3 and TYK2, nor between 3C and PKR and TBK1 in the screenings with the porcine protein library. While there does not appear to be a link between the proteins involved in the above-mentioned non-conserved interactions, STAT2 happens to be one of the targets for which the results of the bovine and porcine screening diverge. Thus, while 3A interacts with the bovine form of STAT2 but not its porcine form, the opposite case is observed for 3C. It would thus be relevant to study in more detail the conservation of this protein between cattle and swine. Even if there are point mutations between the bovine and porcine forms observed using the Constraint-based Multiple Alignment Tool (NCBI), it seems that numerous STAT2 activity domains are conserved (Smart-embl). Thus, the N-terminal domain, which is responsible for protein interactions, the all-alpha helical domain, the DNA-binding domain and the C-terminal domain of STAT2, which allows export of STAT2 into the cytoplasm, do not seem to be determinant in the PPI evidenced with 3A and 3C [62].

Interestingly, IRF3 is one of the proteins for which an interaction could be observed when its porcine form was used, unlike its bovine version, with 3C. While some mutations are noticeable between the bovine and porcine sequences, at the domain tryptophan pentad repeat, which is involved in DNA binding, they do not seem to alter the functioning of this activity domain. Differences in interactions between the bovine and porcine forms could also be demonstrated for TYK2. Indeed, the bovine form was identified as an interactor of 3A and 3D^pol^, but not the swine one. TYK2 was also not found to interact with 3D^pol^ in its ovine and caprine versions. As with most of the proteins focused on in this study, bovine, ovine and caprine forms are highly conserved, whereas divergences appear when ruminant-derived protein sequences are aligned with porcine ones. This could again help to explain the observed differential interactions. However, among the known TYK2 activity domains, all appear to be conserved, namely, the membrane attachment domain, Src homology 2 domains, phosphotransferases and the tyrosine-kinase domain (Smart-embl). Other discrepancies were revealed between the 3D^pol^ polymerase screens performed within our study. For example, MAVS was only shown to interact with 3D^pol^ in its porcine version in the screenings performed. Such differences could be associated with the numerous mutations observed between ruminants and porcine MAVS forms. However, as these mutations do not seem to affect the caspase activation and recruitment domains (CARD), a key region of MAVS activity, other domains that may affect MAVS activity could be involved. The study of the relative importance of the PPI identified through the nanoluciferase screening allowed us to separate the swine model from the ruminant ones. Indeed, among the most important interactors in cattle, sheep and goats, IKKε and IKKα do not seem to be as important in pigs. These discrepancies could be explained by some point mutations between the ruminant and porcine protein sequences. However, these mutations do not seem to have an impact on the known activity domains of these proteins, respectively, tyrosine kinase and serine/threonine kinase. The interactions between the different versions of STAT2 and 3D^pol^ also seem to follow the same pattern. Conversely, according to our study, IRF3 and RIG-I have been shown to be much more important interactors in swine than in ruminants. However, it would appear that IRF7, which is shown as the second-most important interactor for all four species, is involved in a conserved interaction, indicating a possible high importance of this interaction. These interpretations should be taken cautiously, since the relative strength associated with each interaction depends partly on the protein expression efficiency. Furthermore, it is quite possible that a weak interaction may correlate with a significant transient biological function.

The combination of nanoluciferase assays and GST pull-down affinity chromatography enabled the identification and confirmation of numerous interactions involving the 3D^pol^ protein of FMDV O/FRA/1/2001 Clone 2.2. Indeed, we have shown that viral polymerase interacts with bovine, ovine, caprine and swine forms of IKKα, IKKε, IRF3, IRF7 and MDA5, the bovine, ovine and porcine forms of NEMO, and the swine form of MAVS. The performance of the IFN pathway luciferase reporter assays has shown that these PPI, identified at the molecular level, translate into a global phenotypic effect. Thus, it was shown that FMDV 3D^pol^ significantly inhibited the induction phase of the IFN pathway. However, this protein does not appear to have an impact on the amplification phase of the IFN pathway. Although these assays have been conducted in human cells, a model which is not FMDV-susceptible, the resulting observations fully corroborate the findings of the interaction studies, since the 3D^pol^ interactors confirmed by affinity chromatography, IKKα, IKKε, IRF3, IRF7, MDA5 and MAVS, are involved upstream in IFN production.

Given the large number of interactors identified for 3D^pol^ in this study, we would have expected to observe a greater effect on the induction phase of the IFN pathway than the 36% inhibition observed with 3D^pol^ from FMDV O/FRA/1/2001 Clone 2.2 or the 34% observed with 3D^pol^ from FMDV SAT1/KNP/196/91/1 in HEK293T cells. Several hypotheses can be put forward to explain these mild results. On the one hand, it is quite conceivable that not all the protein interactions highlighted in this study act in the same way. Indeed, FMDV is known to either activate or inhibit the immune response depending on what is favourable to its replication. Thus, some interactions could tend towards the activation of the host response, as is the case of the interaction between 2B and LGP2, which enhances the inflammatory response, and others could, in contrast, induce an inhibition of the induced immune response [63]. Moreover, it is possible that not all these interactions are involved in blocking the IFN pathway. On the other hand, if we put ourselves in the global context of infection, FMDV is known to involve almost all of its proteins in fighting against the host cellular response [21,25,26]. It would thus seem to be logical that each protein is not capable, by itself, of inducing a strong inhibition of the immune response, but that the subversion of the immune response induced by FMDV is due to a synergic action between the different viral proteins. It would thus seem extremely interesting to measure the capacities of each of the FMDV proteins, as well as their combined action by performing new luciferase reporter assays. Finally, given the lack of a significant difference in the effect of 3D^pol^ between FMDV O/FRA/1/2001 Clone 2.2 and FMDV SAT1/KNP/196/91/1, which is described as a strain with a high propensity to persist, it does not seem possible to establish a direct relationship between 3D^pol^-mediated inhibition of the IFN pathway and FMDV persistence [64]. In addition, HEK293T cells, non-susceptible to FMDV infection, might not enable the establishment of the interactions identified in our study between the FMDV 3D^pol^ and cellular proteins. This is why we performed luciferase reporter assays in bovine (BUcEC) and porcine (PK-15) cells, which are sensitive to FMDV. By using these cells derived from species of interest, we were able to confirm the tendency observed in HEK293T cells, since a 3D^pol^ inhibitory effect on the IFN response induction phase was observed. This effect is, however, less pronounced than for HEK293T, resulting in respective inhibitions of 27% and 21%. Nevertheless, it is interesting to note that the observed effect is related to the 3D^pol^ and not to the method’s noise, since the overexpression of FMDV 3Bt, for which we did not identify any interaction, did not induce any significant inhibition of the IFN response. As the transfection efficiency associated with these cells is considerably lower than for HEK293T, the results obtained are potentially under-representative of the biological reality. Thus, the induction of interferon response is reduced compared to the HEK293T assays, as well as the inhibition associated with the BTV8 NS3 control. These mild results can also be explained through the fact that our reporter system, like most of those used in the literature, is adapted to human cells and may underperform in other cell models. The challenge of finding cells that are both efficiently transfectable and relevant to the virus underlines the importance of developing suitable in vitro models.

This study has thus shed light on the putative role of FMDV 3D^pol^ in subverting the immune system of its hosts. Until now, this protein, essentially known for its major role in the viral cycle as an RNA-dependent RNA polymerase, has been the subject of very few studies concerning its action against the antiviral response.

Indeed, to date, only two interactions have been described for 3D^pol^, with mouse protein Sam68 [27] and swine ATP-dependent RNA helicase DDX1 [28]. Sam68 is a positive regulator of mRNA stability also targeted by 3C, and DDX1 is a factor involved in both the inhibition of FMDV replication and the production of type-I IFN. While the 3D^pol^–Sam68 interaction has been characterised as a specific binding to prevent Sam68 translocation to the nucleus, both the mode of interaction and the biological significance of 3D^pol^–DDX1 have not been explored, indicating that this newly discovered role of 3D^pol^ is still understudied [27]. Similarly, after having long been side-lined in favour of their importance in viral structure, the impact of FMDV structural proteins in fighting the immune system has recently been shown to be much more important than the scientific community had thought. More generally, the large number of studies on FMDV-associated PPI show that this virus dedicates almost its entire small genome to fighting the immune system and especially the type-I interferon pathway [26,65].

The 3D^pol^ interactors identified in this study are among the IFN pathway proteins involved in more than 75% of virus–host interactions (review in preparation). They are thus major proteins of the IFN pathway, whose subversion is extremely important for FMDV. This is evidenced by the fact that MAVS, a central protein in IFN pathway signalling, is also targeted at the RNA and protein levels via different mechanisms by FMDV VP1, L_pro_ and 3A [44,57,58]. Many other viruses have made MAVS one of the key points in the fight against the antiviral response, including picornaviruses such as HRV-1A, hepatitis A virus (HAV) and SVV, which target MAVS via the 2A and 3C; 2B and precursor 3ABC; and 3C proteins, respectively [66,67,68]. Similarly, NEMO, the protein responsible for the formation of the IKK complex, is also impacted by FMDV 3C, responsible for its swine form cleavage, at the Gln383 residue, to prevent NF-kB activation [49,59,69]. The proteins IRF3 and IRF7, which, after dimerization and translocation into the nucleus, associate with type-I IFN promoter to stimulate their transcription, are all targets of Lpro. Indeed, the FMDV leader protein is responsible for the proteolytic degradation of porcine IRF3 and IRF7 in a dose-dependent manner, but does not affect their mRNA transcription [50]. While no interaction between FMDV and IKKα nor IKKε has been demonstrated to date, this is not the case for other viruses. Indeed, EV71 2C has already been shown to suppress IKKα phosphorylation by recruiting IKKβ and IKKα into viral inclusion bodies [70]. Similarly, encephalomyocarditis virus 3C has been shown to target IKKε through disrupting the TANK-TBK1-IKKε-IRF3 complex to limit IFN I production [71]. Finally, MDA5, which is a cytoplasmic sensor of viral RNA playing a major role in sensing viral infection by recognising long double-stranded RNA, is also targeted by FMDV L_pro_, 2B, 3A and 3C [44,47,48,52]. Interestingly, it turns out that MDA5 is one of the few IFN pathway proteins for which interactions with the 3D^pol^ of other picornaviruses have been demonstrated. Indeed, it has been shown that the 3D^pol^ of coxsackievirus B3 interacts directly with human MDA5 [72]. Similarly, the 3D^pol^ of enterovirus EV71 has also been described as interacting with the human MDA5 CARD domain [72]. Comparison of the protein sequences of coxsackievirus B3 and EV71 3D^pol^ with that of FMDV 3D^pol^ showed few conserved regions. Among these regions, none corresponds to a known motif that could be related to the interaction with MDA5. Therefore, it is not possible to determine at this time whether the FMDV 3D–MDA5 interaction is similar to that of the other two picornaviruses. Other studies concerning picornavirus 3D^pol^ have shown an interaction between EV71 and another important player in the IFN pathway, namely STAT1 [73]. A 3D^pol^–Sam68 poliovirus (PV) interaction could also be demonstrated, a sign of conservation of certain interactions between the viruses of this family [74]. Virus-type-I IFN pathway interactions are also associated with autophagy and apoptosis, as these programmed cell death processes are activated by FMDV to facilitate viral replication and to clear certain components of the IFN pathway [75]. MAVS protein is an interesting example, since FMDV VP0 induces its degradation via the apoptotic pathway [76].

Although globally quite conserved, it is conceivable that the differences in interspecific PPI revealed might contribute to explaining some of the differences observed between cattle, sheep, goats and swine during infection with the same strain of FMDV, such as varying degrees of infectivity, different pathogenesis, and differential persistence [5,6,77]. In general, and as expected from the evolutionary dynamics between ruminants and swine, sequence alignments showed high conservation of type-I IFN pathway proteins among ruminants, whereas considerably more mutations were observed between ruminants and swine. The evidence of an interaction between 3D^pol^ and MAVS in swine, a species unlikely to be persistently infected, but not in ruminants, in which FMDV can persist, could be an opportunity to investigate a potential link between PPI and viral persistence [14,15,16]. Nevertheless, in view of the numerous studies carried out regarding the differential persistence of FMDV, it seems obvious that this phenomenon cannot be based exclusively on a single protein interaction but rather on the establishment of an extremely complex equilibrium involving numerous viral and cellular factors.

## 5. Conclusions

**Figure 10:**
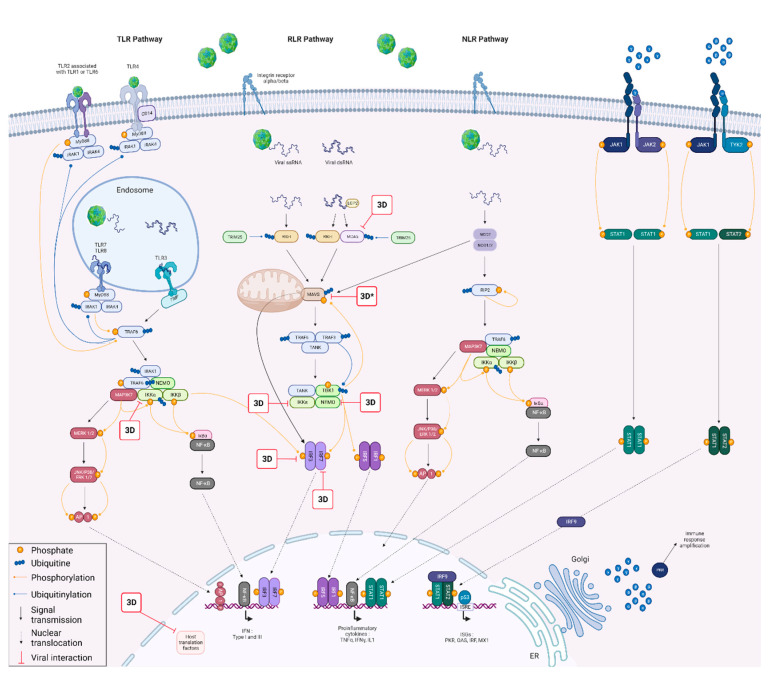
Innate antiviral immune responses and FMDV 3D^pol^ counteraction. The induction of the IFN response results from the recognition of characteristic viral patterns by cellular membrane or cytoplasmic receptors. Activation of these receptors triggers a signalling cascade in the cell, leading to the activation of transcription factors involved in the production of IFN-alpha and IFN-beta and pro-inflammatory cytokines. Finally, the signalling phase consists of the binding of IFN-alpha and beta to their receptors, which generates an activation signal that propagates through the cell via the JAK/STAT pathway to allow the expression of numerous proteins with antiviral or immunomodulatory activity. It has been shown in this study that FMDV 3D^pol^ has an inhibitory effect on the IFN pathway induction phase. This inhibitory effect could be related to its ability to interact in vitro with numerous IFN pathway proteins derived from cattle, sheep, goat and swine, namely, IKKα, IKKε, IRF3, IRF7, MDA5 and NEMO, as well as with the swine version of MAVS (represented by 3D*). Adapted from Sarry et al [26]. Created with BioRender.com (accessed on 11 October 2022).

In conclusion, the present study reveals novel interactions between FMDV 3D^pol^ and type-I IFN pathway proteins from cattle, sheep, goat and swine. These interactions have been represented in a summary scheme (Figure 10), focusing on the putative role of FMDV 3D^pol^ in subverting the immune response. This represents one of the first studies to demonstrate interactions between FMDV polymerase and the host immune response. It is also the first study to show an effect of 3D^pol^ on the major actors of the IFN pathway and, more precisely, an inhibitory effect of the viral protein on the IFN induction phase. Our study reveals that most of the observed interactions are conserved between the species of interest. However, their relative strength was not necessarily conserved from one species to another. Such differences could be considered as a potential partial clue to answer some questions concerning the pathogenesis as well as differential persistence of FMDV and should be investigated under infectious conditions to clarify these PPI biological functions. In view of the PPI recently discovered based on other picornaviruses’ 3D^pol^, it would appear that the identification of PPI is far from reaching a saturation point. It therefore seems essential to continue investigations in order to improve our knowledge of the fine molecular dialogue established between the virus and its hosts. Given the results obtained, it seems extremely important to choose study models that are as close as possible to biological systems, with the ultimate objective of studying these interactions in an infection context.

## Figures and Tables

**Table 1:** Putting into perspective the protein–protein interactions already described in the literature between FMDV proteins and proteins that are part of the sixteen IFN pathway proteins we are focusing on, with regards to our nanoluciferase screening results. N.A. = Information not available No* = Found with Lbpro but not Labpro.

Viral Protein	Cellular Target	Direct or Indirect InterAction?	Study Model	Reference	Found by NanoLuc Approach (Cattle)
2B	MDA5	direct	Human	[52]	No
	RIG-I	direct	Swine	[53]	No
3A	MAVS	direct	Human	[44]	No
	MDA5	direct	Human	[44]	Yes
	RIG-I	direct	Human	[44]	Yes
3B	RIG-I	direct	Swine	[54]	No
3C	MDA5	direct	Swine	[48]	Yes
	STAT1/2	co-factor(s)	Human	[55]	No
	NEMO	direct	Swine	[49]	Yes
	PKR	indirect	Swine	[56]	No
Lb_pro_	MDA5	direct	Human	[47]	Yes
	STAT1/2	direct	Swine	[51]	Yes
	TRAF3	direct	Human	[46]	No
	TBK1	direct	Human	[46]	No
	RIG-I	direct	Human	[46]	No
Lab_pro_	IRF3	direct	Swine	[50]	No*
	IRF7	direct	Swine	[50]	No*
	TBK1	direct	Swine	[57]	No
	MAVS	N.A.	Swine	[57]	No
VP1	MAVS	direct	N.A.	[58]	No
VP3	MAVS	direct	Human	[59]	No

## Data Availability

All data are given in the Results section.

## Supplementary Materials

The following supporting information can be downloaded at: https://www.mdpi.com/1999-4915/15/3/666/s1, Table S1: Primers used for viral sequence amplification; Table S2: IFN pathway protein Genbank references; Figure S1: nanoluciferase screenings against the bovine library; Figure S2: nanoluciferase screenings against the swine library; Figure S3: GST pull-down analysis of PPI between FMDV 3D^pol^ and cattle, sheep, and goat protein libraries.
