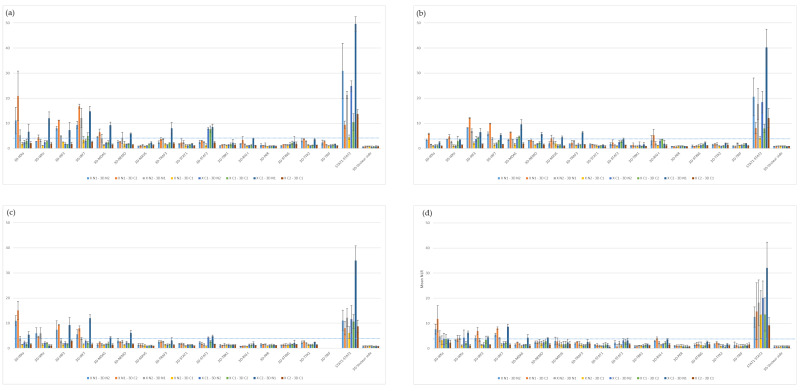# Correction: Sarry et al. Host-Specific Interplay between Foot-and-Mouth Disease Virus 3D Polymerase and the Type-I Interferon Pathway. *Viruses* 2023, *15*, 666

**DOI:** 10.3390/v15112137

**Published:** 2023-10-24

**Authors:** Morgan Sarry, Grégory Caignard, Juliette Dupré, Stephan Zientara, Damien Vitour, Labib Bakkali Kassimi, Sandra Blaise-Boisseau

**Affiliations:** 1 UMR VIROLOGIE, INRAE, École Nationale Vétérinaire d’Alfort, ANSES Laboratoire de Santé Animale, Université Paris-Est, 94700 Maisons-Alfort, France; 2 AgroParistech, 16 Rue Claude Bernard, 75005 Paris, France

## Error in Figure

In the original publication [1], there was a mistake in “Figure 4. Screening for protein–protein interactions between FMDV 3Dpol and the sixteen selected IFN pathway proteins from the cattle, sheep, goat and swine libraries.” as published. **The graphs had been switched with those in Figure S1**. The corrected “Figure 4. Screening for protein–protein interactions between FMDV 3Dpol and the sixteen selected IFN pathway proteins from the cattle, sheep, goat and swine libraries.” version appears below. The authors state that the scientific conclusions are unaffected. This correction was approved by the Academic Editor. The original publication has also been updated.

**Figure:**