# Supplementary figures and images for: Host-Specific Interplay between Foot-and-Mouth Disease Virus 3D Polymerase and the Type-I Interferon Pathway
Source: Viruses. 2023 Mar 1;15(3):666. doi: 10.3390/v15030666 (PMC10054395; doi:10.3390/v15030666)

(a)

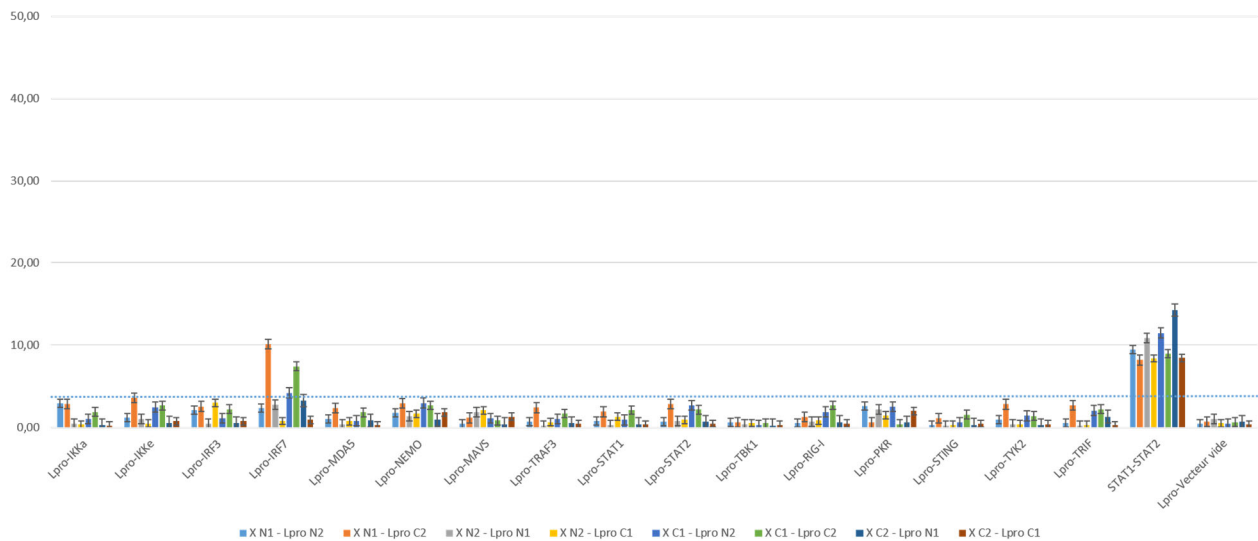

(b)

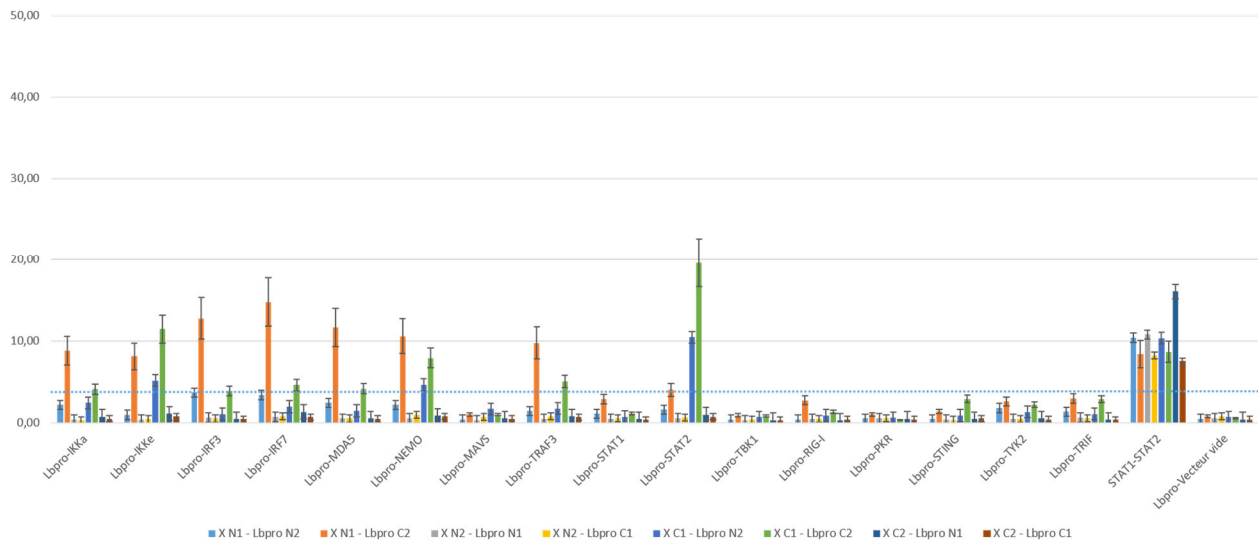

(c)

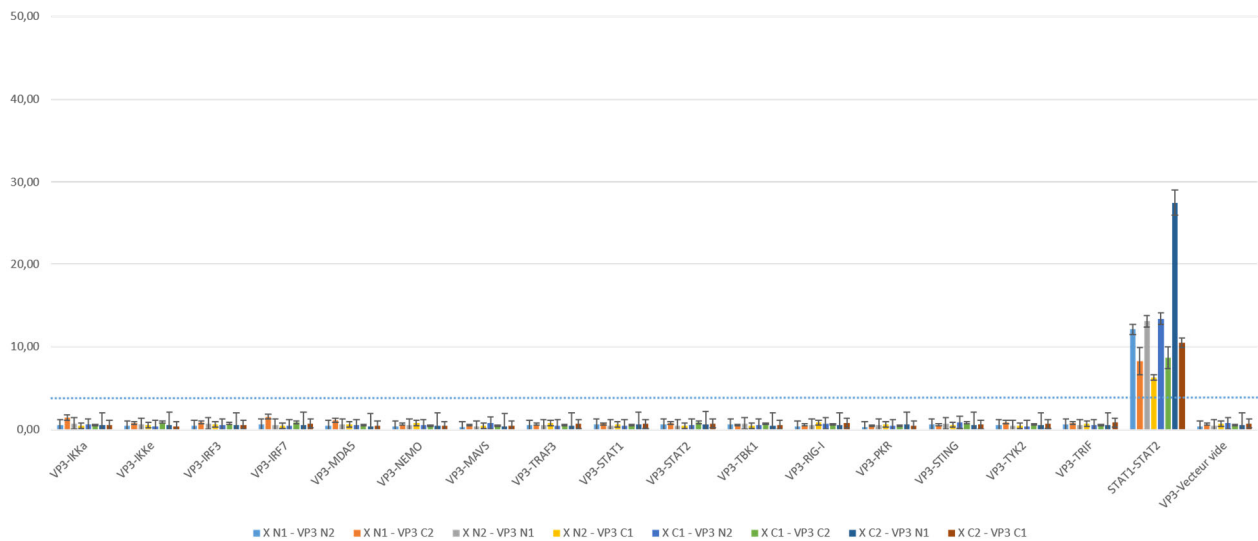

(d)

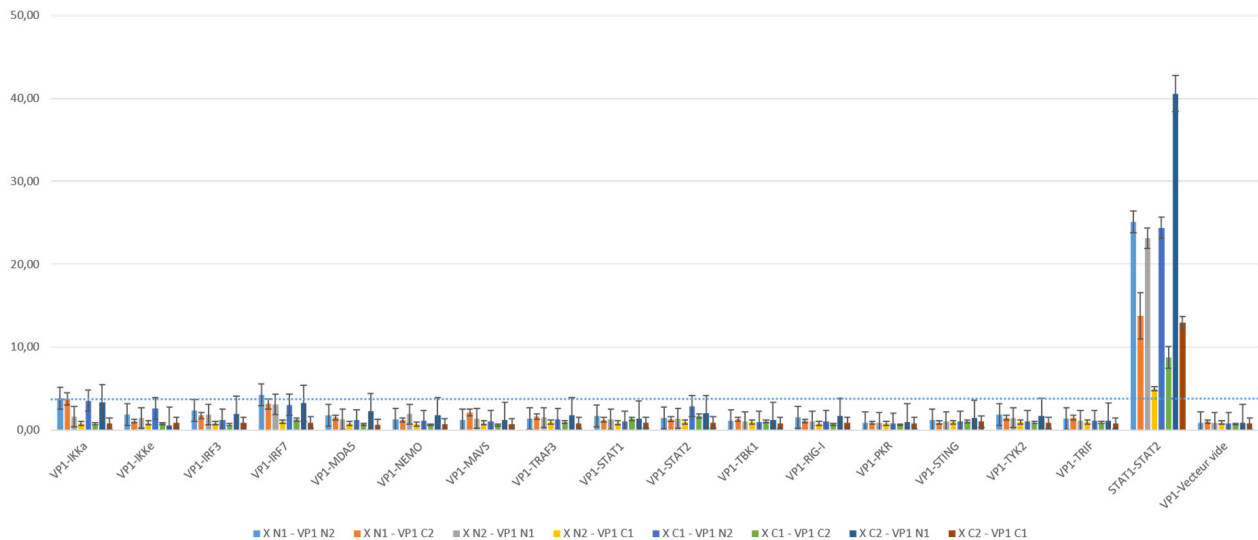

(e)

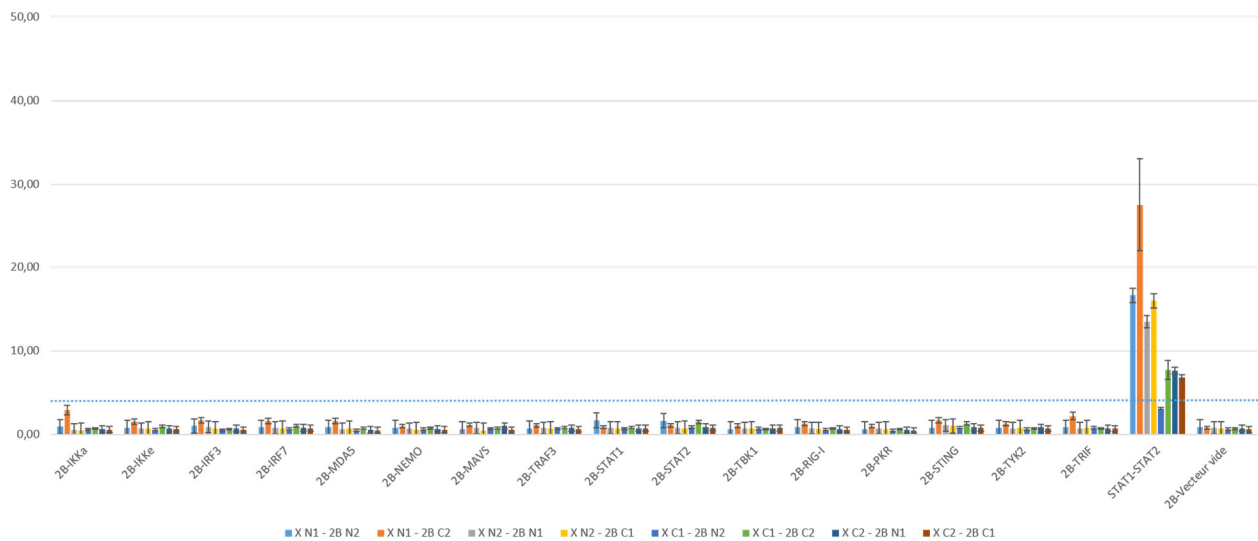

(f)

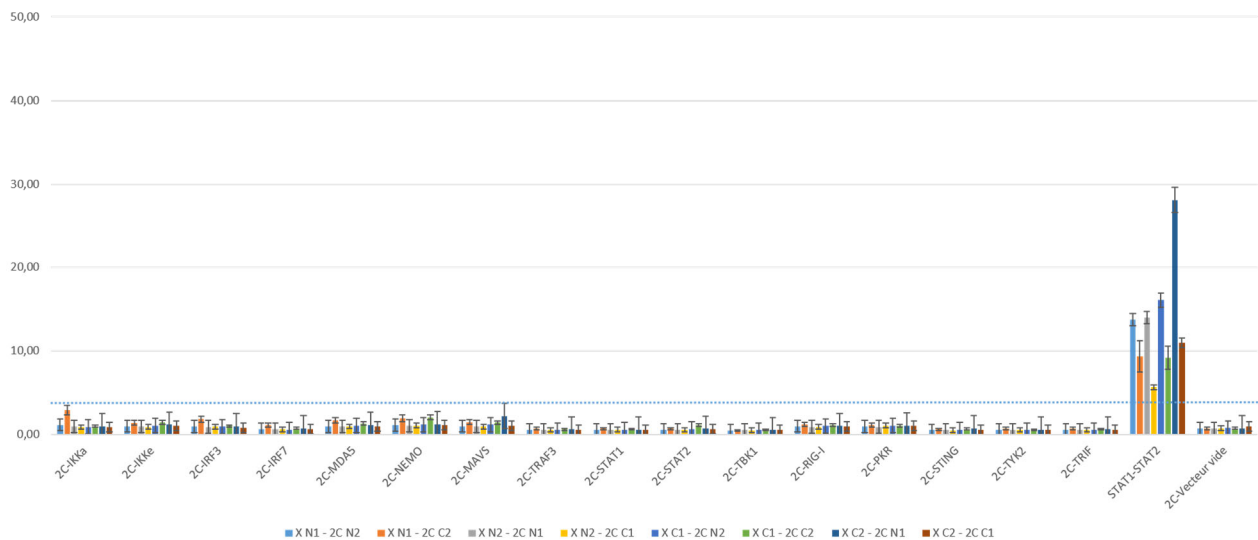

(g)

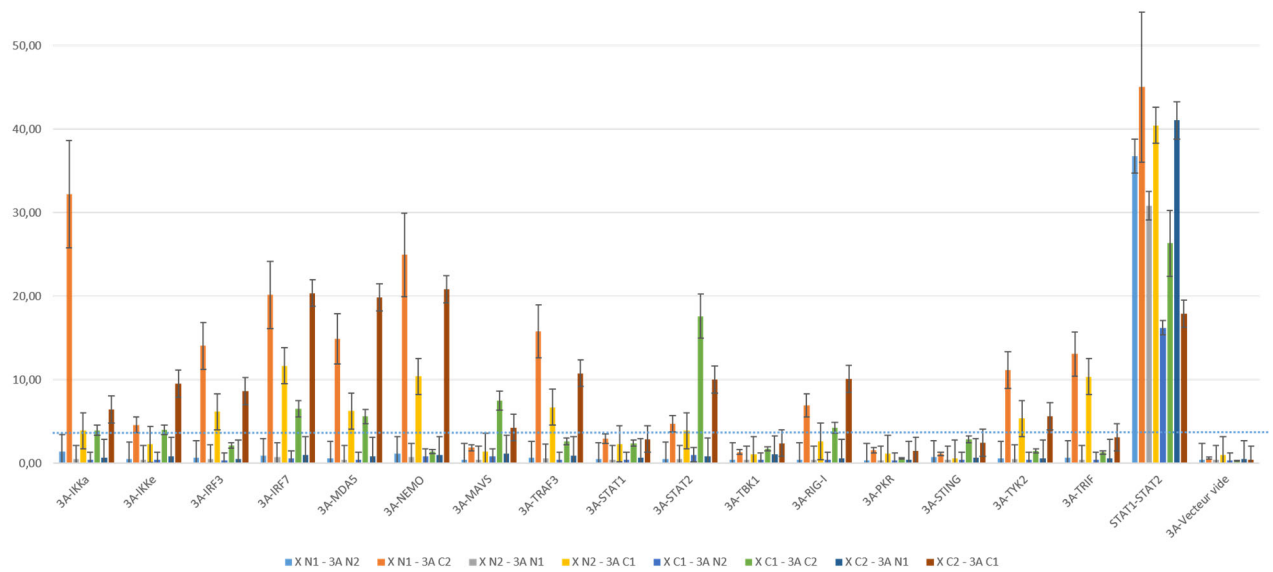

(h)

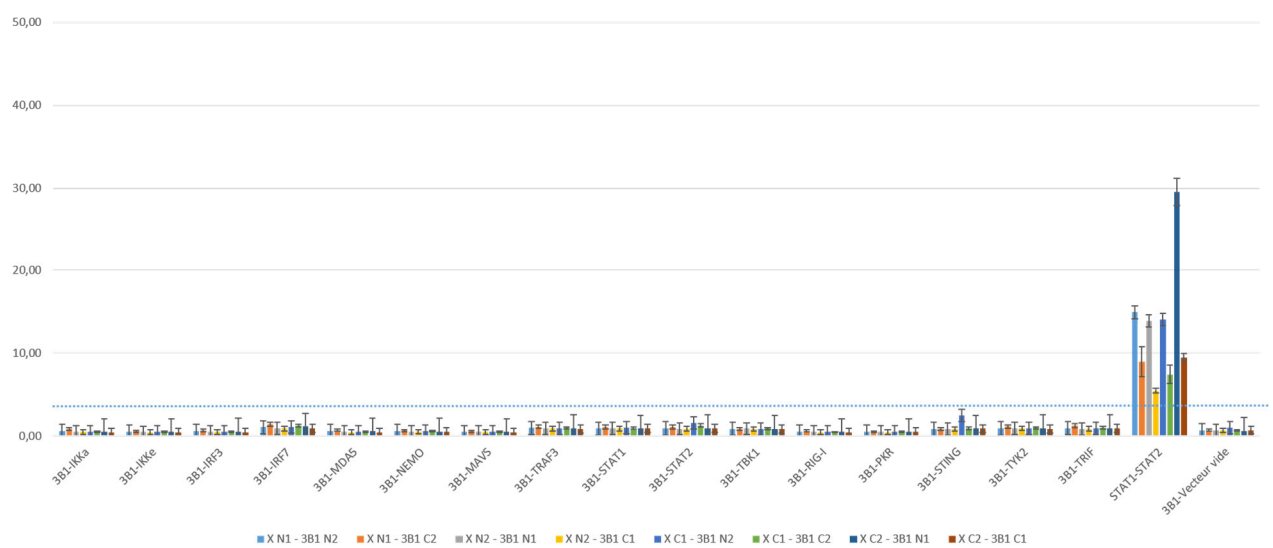

(i)

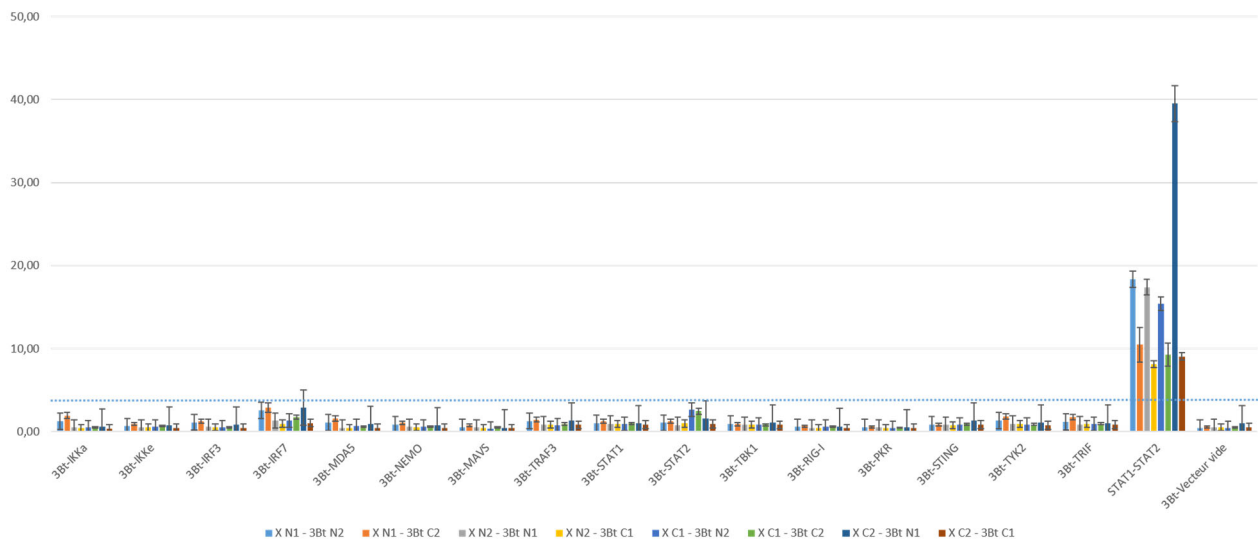

(j)

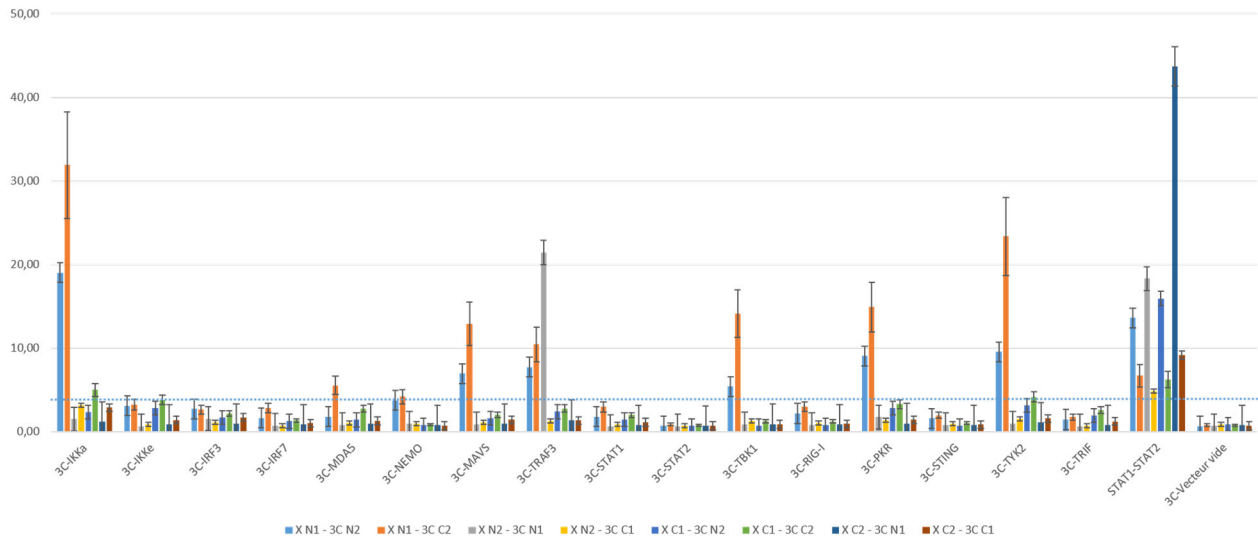

Supplement: Supplementary file 1 [file viruses-15-00666-s001.zip › Figure S1-NanoLuciferase screenings against bovine library.pdf]

(a)

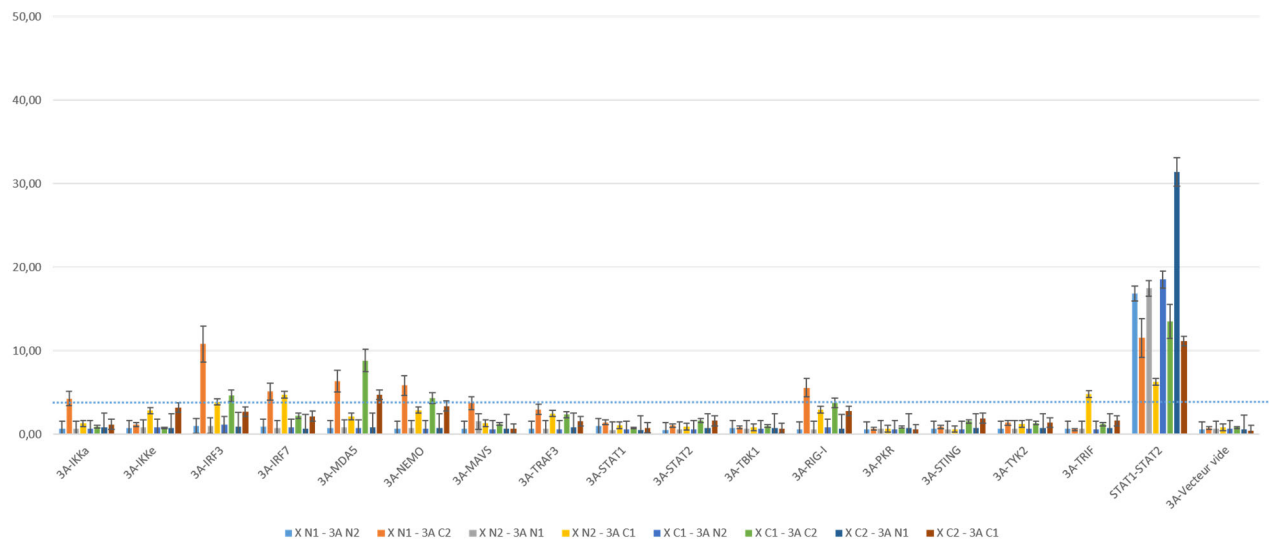

(b)

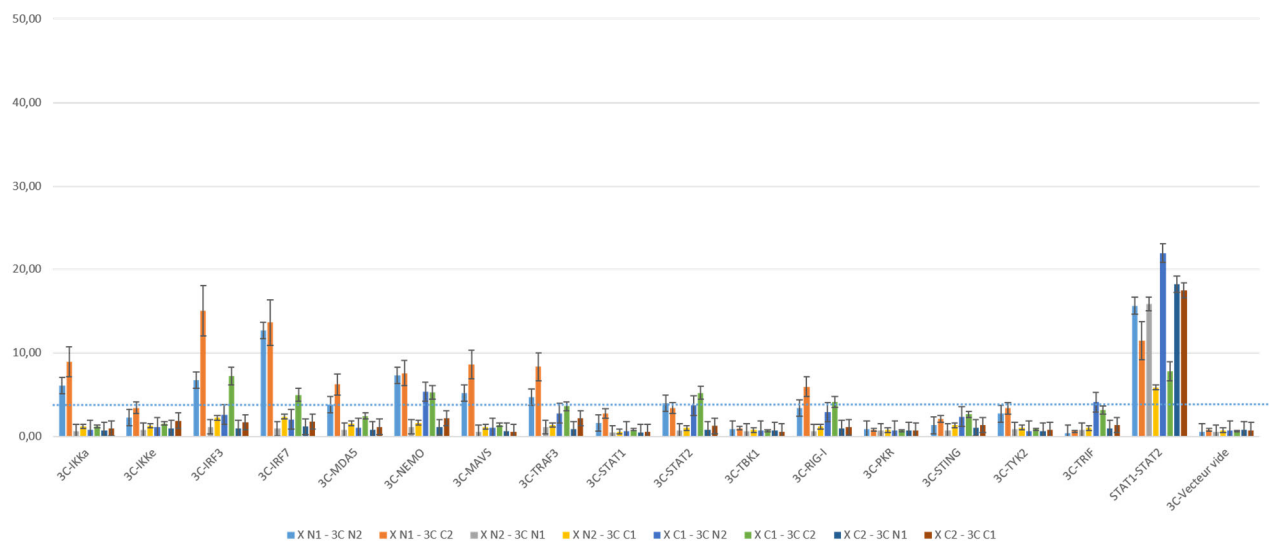

Supplement: Supplementary file 1 [file viruses-15-00666-s001.zip › Figure S2-NanoLuciferase screenings against swine library.pdf]

## Slide 1
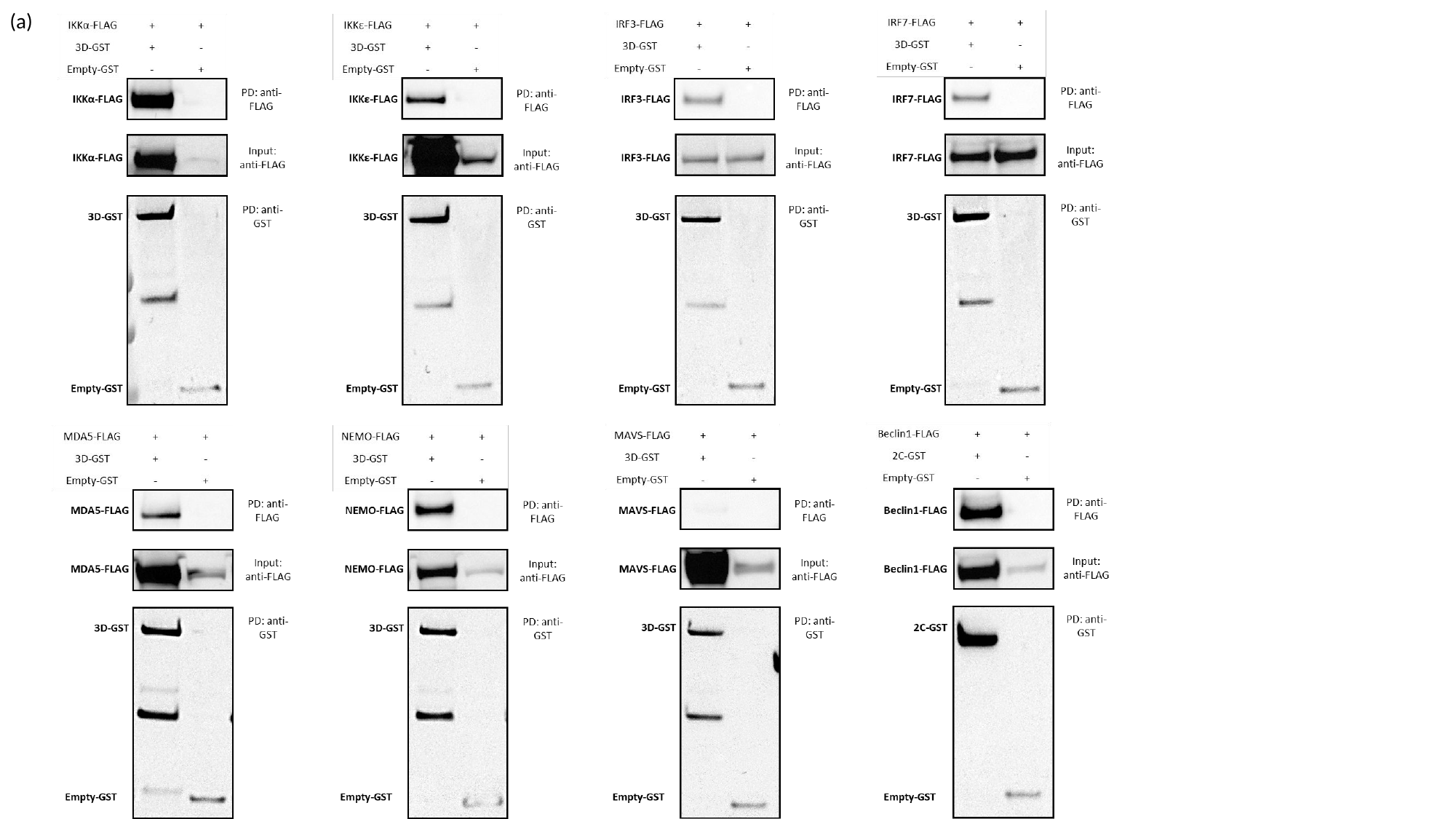

(a)

## Slide 2
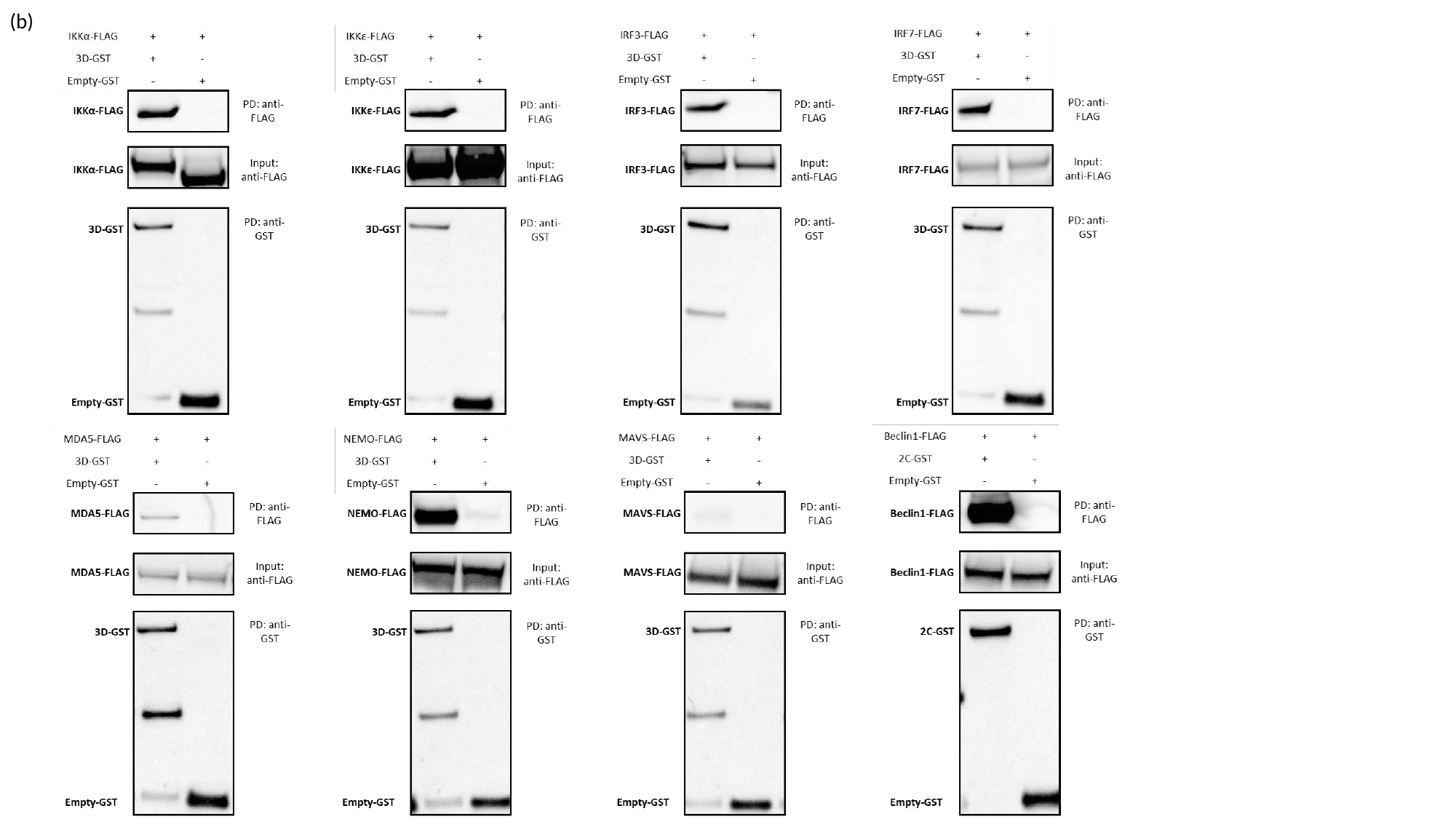

(b)

## Slide 3
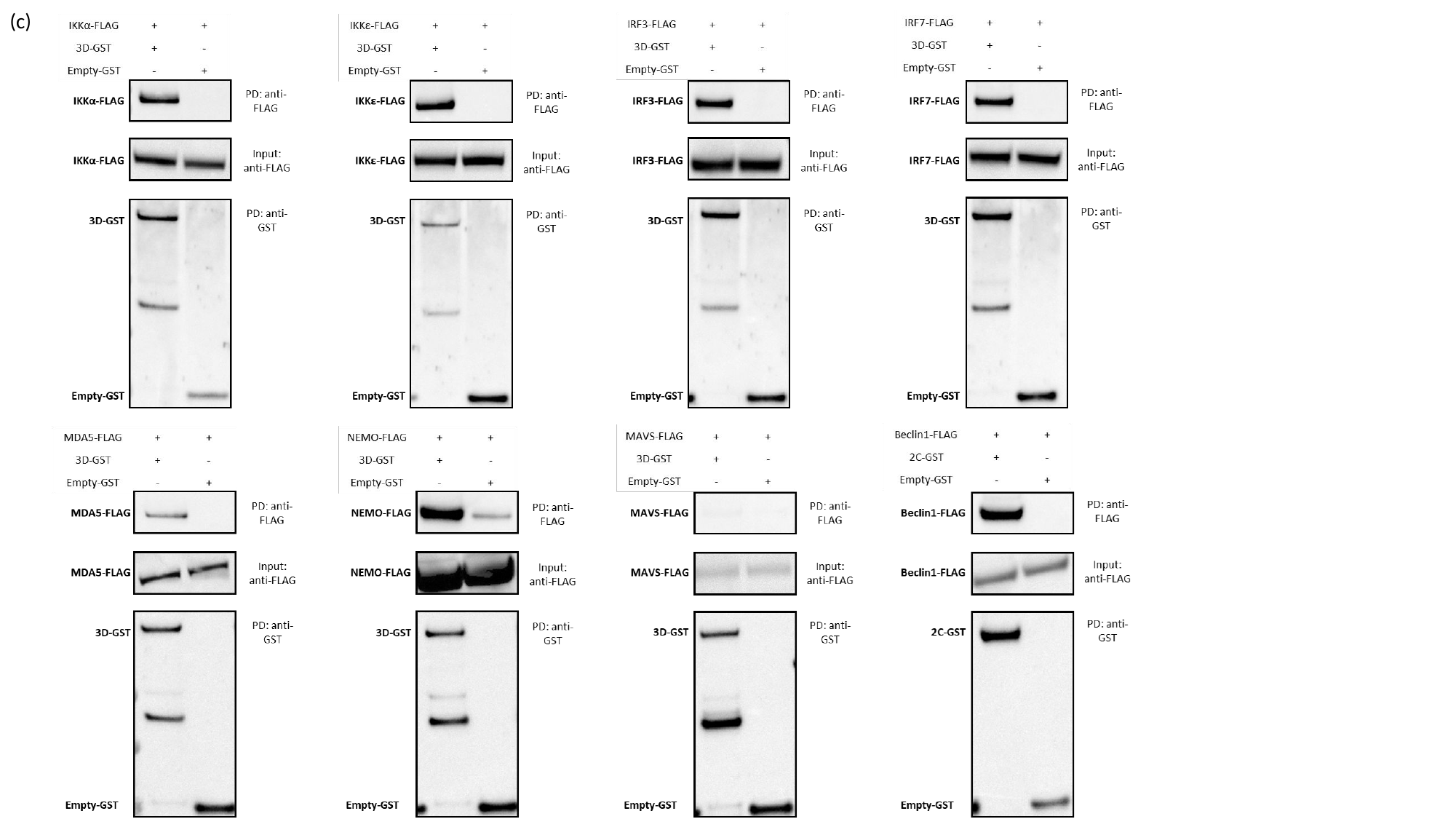

(c)

Supplement: Supplementary file 1 [file viruses-15-00666-s001.zip › Figure S3-GST Pull-down analysis on PPI between FMDV 3D and cattle, sheep, and goat protein libraries.pptx]
